# Functional Effects of *let-7g* Expression in Colon Cancer Metastasis

**DOI:** 10.3390/cancers11040489

**Published:** 2019-04-06

**Authors:** Che-Mai Chang, Henry Sung-Ching Wong, Chien-Yu Huang, Wen-Li Hsu, Zhi-Feng Maio, Siou-Jin Chiu, Yao-Ting Tsai, Ben-Kuen Chen, Yu-Jui Yvonne Wan, Jaw-Yuan Wang, Wei-Chiao Chang

**Affiliations:** 1 Master Program for Clinical Pharmacogenomics and Pharmacoproteomics, School of Pharmacy, Taipei Medical University, Taipei 110, Taiwan; awefld@gmail.com (C.-M.C.); miningyue@gmail.com (H.S.-C.W.); 2 Department of Clinical Pharmacy, School of Pharmacy, Taipei Medical University, Taipei 110, Taiwan; 3 Ph.D. for Medical Biotechnology Program, College of Medical Science and Technology, Taipei Medical University, Taipei 110, Taiwan; 4 Department of Surgery, School of Medicine, College of Medicine, Taipei Medical University, Taipei 110, Taiwan; cyh@tmu.edu.tw; 5 Division of General Surgery, Department of Surgery, Shuang Ho Hospital, Taipei Medical University, Taipei 235, Taiwan; 6 Research Organization for Nano & Life Innovation, Waseda University, Tokyo 162-8480, Japan; hsuwenli0626@gmail.com; 7 Emerging Compounds Research Center, Department of Environmental Science and Engineering, College of Engineering, National Pingtung University of Science and Technology, Pingtung 91201, Taiwan; 8 Department of Medical Research, E-DA Hospital, I-Shou University, Kaohsiung 84001, Taiwan; 9 Division of Colorectal Surgery, Department of Surgery, Kaohsiung Medical University Hospital, Kaohsiung Medical University, Kaohsiung 807, Taiwan; R980063@kmu.edu.tw; 10 Department of Medical Genetics, College of Medicine, Kaohsiung Medical University, Kaohsiung 807, Taiwan; SJChiu82@gmail.com (S.-J.C.); ytt023019@gmail.com (Y.-T.T.); 11 Department of Pharmacology, College of Medicine, National Cheng Kung University, Tainan 701, Taiwan; bkchen58@mail.ncku.edu.tw; 12 Department of Medical Pathology and Laboratory Medicine, University of California, Davis, Sacramento, CA 95817, USA; yjywan@ucdavis.edu; 13 Department of Surgery, Faculty of Medicine, College of Medicine, Kaohsiung Medical University, Kaohsiung 807, Taiwan; 14 Graduate Institute of Medicine, College of Medicine, Kaohsiung Medical University, Kaohsiung 807, Taiwan; 15 Graduate Institute of Clinical Medicine, College of Medicine, Kaohsiung Medical University, Kaohsiung 807, Taiwan; 16 Department of Pharmacy, Taipei Medical University-Wanfang Hospital, Taipei 116, Taiwan; 17 Department of Medicine Research, Taipei Medical University-Shuang Ho Hospital, New Taipei City 235, Taiwan

**Keywords:** colorectal cancer, microRNA, *let-7g*, the cancer genome atlas (TCGA), store-operated calcium (SOC)

## Abstract

MicroRNA regulation is crucial for gene expression and cell functions. It has been linked to tumorigenesis, development and metastasis in colorectal cancer (CRC). Recently, the *let-7* family has been identified as a tumor suppressor in different types of cancers. However, the function of the *let-7* family in CRC metastasis has not been fully investigated. Here, we focused on analyzing the role of *let-7g* in CRC. The Cancer Genome Atlas (TCGA) genomic datasets of CRC and detailed data from a Taiwanese CRC cohort were applied to study the expression pattern of *let-7g*. In addition, in vitro as well as in vivo studies have been performed to uncover the effects of *let-7g* on CRC. We found that the expression of *let-7g* was significantly lower in CRC specimens. Our results further supported the inhibitory effects of *let-7g* on CRC cell migration, invasion and extracellular calcium influx through store-operated calcium channels. We report a critical role for *let-7g* in the pathogenesis of CRC and suggest *let-7g* as a potential therapeutic target for CRC treatment.

## 1. Introduction

Micro (mi)RNAs are short, single-stranded non-coding RNAs which function as post-transcriptional regulators of gene expression and are involved in fundamental biological processes. First identified from developmental timing in the model animal *Caenorhabditis elegans*, miRNA is well-known for conserved properties among different species [1]. Recently, many studies have been published showing that miRNAs are involved in critical human diseases, including cancer [2,3,4]. miRNAs suppress gene expression through imperfect base-pairing with a complementary sequence in the 3′-untranslated region (UTR) of target messenger (m)RNAs and then inhibit target protein synthesis by preventing translation or inducing mRNA degradation [5,6]. The biological roles of miRNAs are as regulators of several cellular events, such as proliferation, differentiation and apoptosis, which are associated with cancer progression [6,7]. For instance, *miR-21* mediates the post-transcriptional modification of a tumor suppressor, thereby stimulating colorectal cancer (CRC) invasion, intravasation and metastasis [8]. Ma et al. stated that *miR-9* causes E-cadherin downregulation and activates β-catenin signaling to enhance the level of vascular endothelial growth factor (VEGF), which in turn promotes breast tumor angiogenesis [9].

The *let-7* family, one of the first discovered miRNAs, functions as key regulators in development and cancer [10,11]. During *C. elegans* development, *let-7* is present in the larval to adult stages and causes seam cells to stop dividing and differentiate [12]. In *let-7* mutant nematodes, seam cells fail to exit the cell cycle, leading to continuous seam cell division and the death of the worms, so the name *lethal-7* was given to it after this discovery [11]. There are 13 kinds of *let-7* in humans, among which *let-7g* has been proven to be tightly associated with hepatocellular carcinoma (HCC), non-small cell lung tumors, breast cancer and CRC [13,14,15,16,17,18]. Previous studies observed the reduction of *let-7g* in tumor tissues of metastatic HCC patients. Overexpression of *let-7g* suppressed HCC cell migration, invasion and proliferation via regulating type I collagen α2 (COL1A2), c-Myc and p16 [15,19]. Other studies demonstrated that expression of *let-7g* was significantly reduced in tumor tissues of breast cancer patients and even lower in those with lymph node metastasis [16,20]. These studies further unveiled inhibitory effects of *let-7g* on breast cancer cell metastasis and proposed that estrogen and epidermal growth factor (EGF) promote breast tumor cell migration and invasion by suppression of *let-7g* through 44/42 mitogen-activated protein kinase (MAPK) signaling activation.

CRC is the third leading cause of cancer deaths around the world [21]. Around 25% of CRC patients suffer from metastatic events, and yet the mechanism of CRC metastasis has not been fully explored due to complex environmental and genomic factors [22,23,24]. The study of let-7 miRNAs could be a possible route to understanding the mechanism of tumor progression and metastasis. Nakajima et al. previously unveiled a significant increase of *let-7g* level in tumor tissues of CRC patients and its association with the poor chemo-response and progressive disease of CRC [13]. Wang et al. further observed that the expression level of *let-7g* was significantly higher in circulating blood samples from CRC patients than in those from healthy individuals [25]. Although *let-7g* was found to be involved in tumorigenesis of CRC by these studies, whether the functional effects of *let-7g* have special roles in colon cancer progression and metastasis needs further investigation based on a systematic and comprehensive study.

To address the questions, applied integrated bioinformatics approaches, functional validation using different CRC samples cohort sets, as well as in vitro and in vivo experiments were conducted. We identified *let-7g*-associated clinical features, transcriptomic profiles and biological pathways in a large CRC cohort from The Cancer Genome Atlas (TCGA) database. Reduced *let-7g* levels in tumor tissues compared to adjacent normal tissues was observed in a Taiwanese population CRC cohort. Functional studies based on in vitro and in vivo approaches further revealed suppressive effects of *let-7g* on CRC cell migration, invasion and store-operated calcium (SOC) influx. In summary, our results provided clinicopathological significance for *let-7g* and in CRC progression, and implicated a role for store-operated Ca^2+^ entry in *let-7g*-mediated CRC metastasis. *let-7g* could be of great pharmaceutical interest for anti-cancer drug discovery.

## 2. Results

### 2.1. Correlation between let-7g Expression and Clinical Profiles in CRC Patients

**Figure 1:**
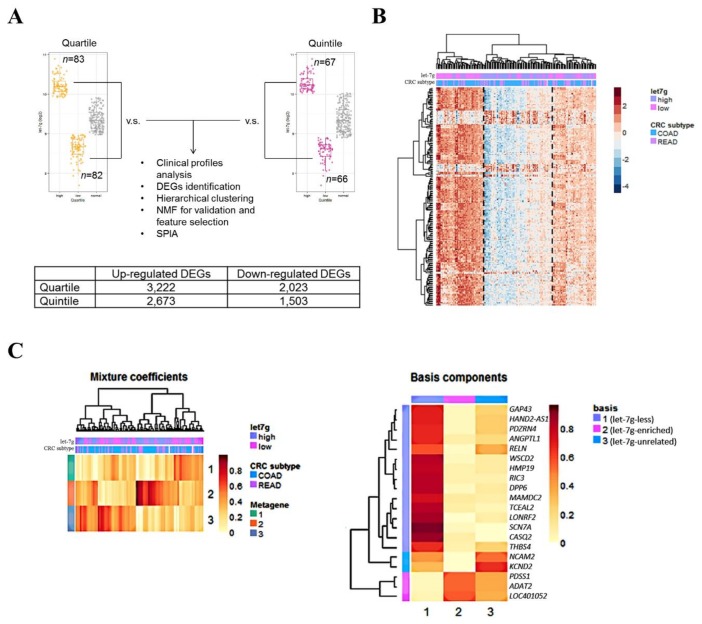

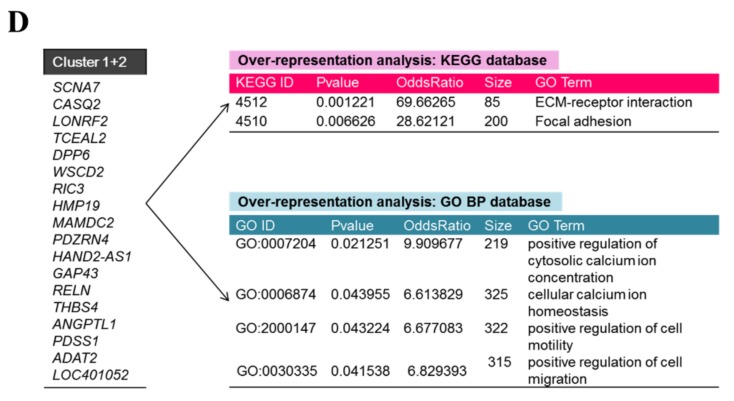
Transcriptomic profiles and functional annotations correlated with *let-7g* overexpression. (**A**) Schematic showing patient categorization of The Cancer Genome Atlas (TCGA) colorectal cancer (CRC) samples. CRC samples were stratified into two groups (overexpression (high) and low-expression (low)) based on the expression value (−log_2_(RPM)) of *let-7g*. Two categorization criteria (quartiles and quintiles) were applied to assess the robustness of the results and were used for a downstream exploratory analysis (upper panel). Numbers of significantly upregulated and downregulated differentially expressed genes (DEGs) based on quartile and quintile categorization are shown (lower panel). (**B**) Heatmap of the top 200 most variant DEGs based on quartile categorization in the RNA sequencing profile. Each column represents CRC samples, and each row represents genes. Two-way hierarchical clustering was performed, and the information of the *let-7g* expression status and the CRC location were mapped. (**C**) Non-negative matrix factorization was performed on the top 200 most variant differentially expressed genes (DEGs) based on quartile categorization. Heatmaps of the metagene matrix constructed with 20 genes were obtained from a factorization rank of 3. (**D**) Of these, 18 genes which were positively correlated to clusters 1 and 2 were used to assess the over-represented Kyoto Encyclopedia of Genes and Genomes (KEGG) pathways and gene ontology (GO) biological process (BP) terms.

TCGA CRC samples with available *let-7g* RNA-sequencing data were categorized into *let-7g* high-expression and *let-7g* low-expression groups based on two criteria: quartiles (*n* = 165) and quintiles (*n* = 133, Figure 1A, upper panel). Based on the quartile categorization criteria, we conducted correlation analysis between dichotomized *let-7g* expression statuses and clinical profiles (Table S1). Results showed a correlation between *let-7g* expression and lymphatic invasion (*p* = 0.027, adjusted *p* = 0.07). Notably, the expression status of *let-7g* showed a negative correlation with the lymphatic invasion status, with 50 of 92 (54.3%) patients without lymphatic invasion belonging to the *let-7g* overexpression group, while 17 of 49 (34.7%) patients with lymphatic invasion belonging to the *let-7g* overexpression group. In contrast, although trends of a negative correlation of the *let-7g* expression status with lymph node metastasis, distant metastasis, disease stage and vascular invasion were observed, none of them reached statistical significance (*p* < 0.05). These results imply a tumor suppressive role of *let-7g* in CRC.

### 2.2. Let-7g-Associated Transcriptomic Profiles Reveal Three CRC Subpopulations

Eighty-three *let-7g* high-expression and 82 *let-7g* low-expression samples based on quartile categorization and 67 *let-7g* high-expression and 66 *let-7g* low-expression samples based on quintile categorization were subjected to an RNA-sequencing profile analysis to identify differentially expressed genes (DEGs) associated with the *let-7g* expression status. We used a moderated *t*-test to identify significant DEGs in each categorization. In total, 3222 (quartile) or 2673 (quintile) upregulated DEGs and 2023 (quartile) or 1503 (quintile) downregulated DEGs were identified and fulfilled the Benjamini and Hochberg (BH)-adjusted *p*-value criteria of <0.05 among the *let-7g* high-expression group and *let-7g* low-expression group, respectively (Figure 1A, lower panel). We noted that the number of identified DEGs in the quintile categorization was smaller than that of the quartile categorization; this was because the quintile categorization can be considered as a more-stringent criteria which included a smaller number of samples.

To explore the aggregative effects of CRC samples based on *let-7g*-associated profiles, we further conducted an unsupervised hierarchical clustering analysis based on the identified DEGs. A heatmap of the top 200 most significant DEGs based on the quartile categorization was visualized (Figure 1B), and a three-group gathering configuration was observed, revealing three highly clustered sample-based within-group expression patterns. To validate this exploratory result, we further conducted the same clustering analysis based on the top 100 and 300 DEGs in the quartile categorization, and results suggested that the three-group gathering configuration was also distinguishable (Figure S1A). The analysis focused on the top 100, 200 and 300 DEGs based on the quintile categorization also showed the same three-group gathering pattern (Figure S1B). Based on these results, three sample groups were identified: a group enriched in *let-7g* overexpression patients, which was correlated with downregulation of most of the top DEGs (*let-7g*-enriched); a group enriched in *let-7g* low-expression patients, which was correlated with upregulation of most of the top DEGs (*let-7g*-less); and a group composed of similar proportions of patients of *let-7g* overexpression and low-expression, which showed no significant correlation with the top DEGs (*let-7g*-unrelated).

To validate the robustness of the clusters identified, non-negative matrix factorization (NMF) was carried out against the top 100, 200 and 300 DEGs in both the quartile and quintile categorization. We adopted the Brunet algorithm to perform the NMF analysis on the factorization rank between 2 and 6 in 200 runs. As expected, all results showed that the optimal number of clusters was three, which showed the strongest consensus signature (Figures S2 and S3).

Notably, when CRC samples were stratified into three groups based on NMF, the *let-7g*-less group (cluster 1) had a significantly higher incidence of advanced-stage tumors (FDR adjusted *p* = 0.038) and lymphatic invasion (FDR adjusted *p* = 0.044) compared to the *let-7g*-enriched group (cluster 2) and *let-7g*-unrelated group (cluster 3, Table 1).

### 2.3. Targets of let-7g were Significantly Enriched in let-7g-Associated Profiles

To further assess the biological significance of the characterized *let-7g*-associated profiles, we first queried *let-7g* targets using the TarBase (v7.0) database. Then, we tested whether genes in the target list showed enrichment for association (*p*-value) and magnitude (fold-change) signals. Intriguingly, the *let-7g* targets showed highly significant enrichment for association signal (*p* = 9.99 × 10^−5^) and magnitude signal (*p* = 3 × 10^−4^) in comparison with non-target genes based on quartile categorization. Similar results (association signal (*p* = 9.99 × 10^−5^) and magnitude signal (*p* = 4.1 × 10^−4^)) were also observed based on quintile categorization (Figure S4).

### 2.4. Prospecting let-7g-Associated Biological Features in CRC Patients

To identify the underlying biological signatures correlated with *let-7g* expression, we next extracted the most important DEGs from *let-7g*-associated profiles and conducted the over-representation analysis for these genes. We first extracted 20 DEGs as selected features for metagenes specific to three *let-7g*-associated CRC subtypes identified as previously described based on a NMF-based feature extraction approach (Figure 1C). We further selected 18 metagene-specific DEGs from the *let-7g*-less group (cluster 1) and the *let-7g*-enriched group (cluster 2) as the most important *let-7g*-associated DEGs for investigating relevant biological features (Figure 1D). Two significant over-represented Kyoto Encyclopedia of Genes and Genomes (KEGG) pathways were identified: extracellular matrix (ECM)-receptor interaction and focal adhesion, which are known to be correlated with tumor aggressiveness and metastasis. In addition, based on the results of the gene ontology (GO) biological process (BP) analysis, we observed several over-represented terms related to calcium ion signaling and cell motility (Figure 1D), including positive regulation of the cytosolic calcium ion concentration (GO:0007204), cellular calcium ion homeostasis (GO:0006874), positive regulation of cell motility (GO:2000147), and positive regulation of cell migration (GO:0030335). Other significantly over-represented GO BP terms are shown in Table S2.

To gain biological insights into the transcriptomic regulatory role of *let-7g*, we conducted a signaling pathway impact analysis (SPIA) based on quartile categorization criteria. Significant pathways (*p* = 2 × 10^−6^) are listed in Table S3. The focal adhesion pathway (Figure S5), calcium signaling pathway (Figure S6), and ECM-receptor interaction pathway (Figure S7), which were consistent with the results of over-representation analysis (ORA), showed significant negative perturbation evidence. In addition, cytokine- and chemokine-related pathways were also significantly negatively perturbed (Figures S8 and 9). Specifically, SPIA results revealed a significant negative perturbation of pathways in cancer (Figure S10), suggesting a comprehensive negative impact of *let-7g* overexpression on cancers.

### 2.5. Reduced let-7g in CRC Specimens

**Figure 2:**
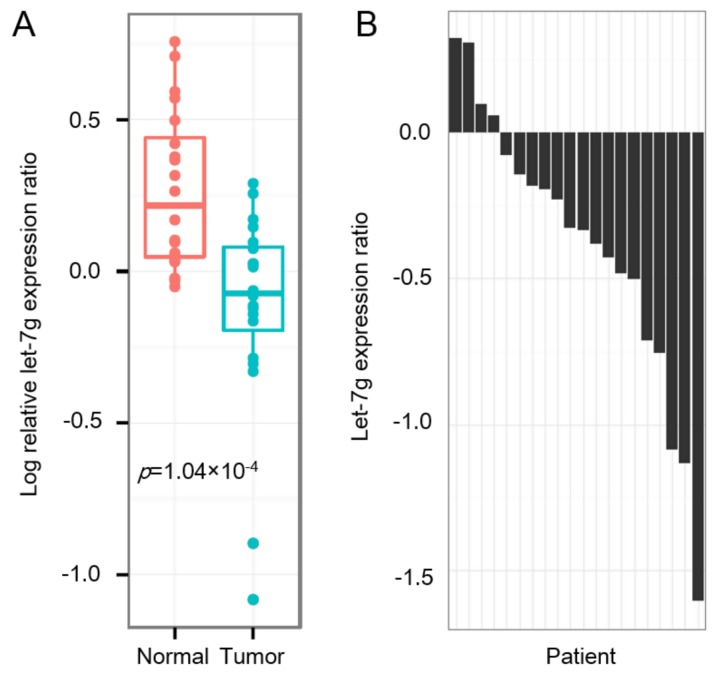
Expression of *let-7g* in a cohort of colorectal cancer (CRC) patients. (**A**) Whisker boxplot shows *let-7g* mRNA values of tumor tissues and paired adjacent normal tissues in 20 CRC samples. (**B**) Bar plot shows the relative expression ratio between *let-7g* from tumor versus paired normal tissues around the cancer tissue of each CRC patient.

To further investigate the role of *let-7g* in CRC, we quantified the level of *let-7g* in 20 CRC patients. Real-time qPCR data showed that the level of *let-7g* was significantly lower in cancer specimens in comparison with that of adjacent benign tissues (Wilcoxon rank sum *p* = 1.04 × 10^−4^). The expression ratio of *let-7g* in the CRC cohort was calculated by tumor versus normal tissues. As shown in Figure 2B, 16 of 20 (80%) CRC patients showed decreased expression of *let-7g* in tumor tissues compared to paired adjacent normal tissues. In addition, we also investigated the clinicopathological role of *let-7g* in a Taiwanese CRC cohort. However, none of the clinical parameters showed a significant correlation with *let-7g* expression levels (Table S4).

### 2.6. Let-7g Inhibits CRC Cell Migration in Vitro

**Figure 3:**
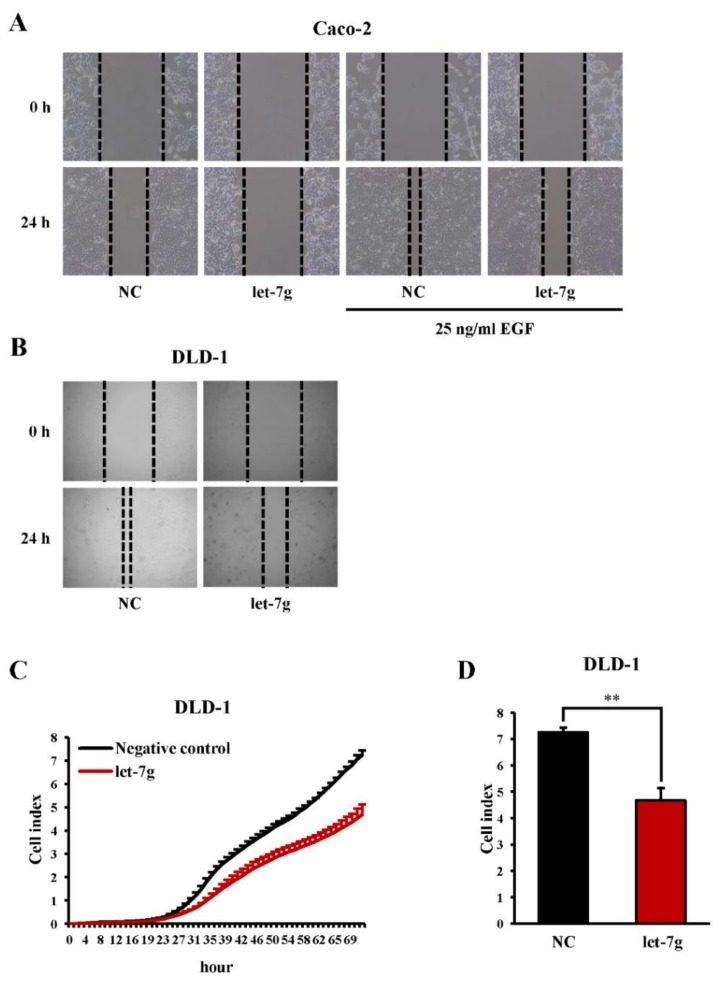
Effect of *let-7g* on cell migration in colorectal cancer cells. (**A**,**B**) A cell-free gap was generated by seeding a miRNA negative control and *let-7g*-transfected cells inside a culture insert to perform a wound-healing assay. The cell migration of Caco-2 (magnification, ×10) and DLD-1 (magnification, ×20) cells with and without epidermal growth factor (EGF) treatment was assessed by imaging at 0- and 24-h time points. (**C**) The real-time cell migration of the miRNA negative control and *let-7g*-transfected DLD-1 cells was analyzed by an x’Celligence Biosensor System. (D) The real-time DLD-1 cell migration after 72 h was quantified. **, statistically significant at a *p*-value of <0.01.

We tested whether *let-7g* affects the migration of colon cancer cells. As shown in Figure 3A, EGF-induced cell migration of Caco-2 cells was diminished by *let-7g* in a wound-healing assay. Additionally, we also observed an inhibitory effect of *let-7g* on DLD-1 cell migration in both the wound-healing assay and biosensor experiment for migration (Figure 3B–D). Accordingly, our results showed that *let-7g* was capable of inhibiting CRC cell migration.

### 2.7. Both let-7g and the Store-Operated Calcium (SOC) Channel Blocker SKF96365 Inhibit CRC Cell Motility in Vitro

**Figure 4:**
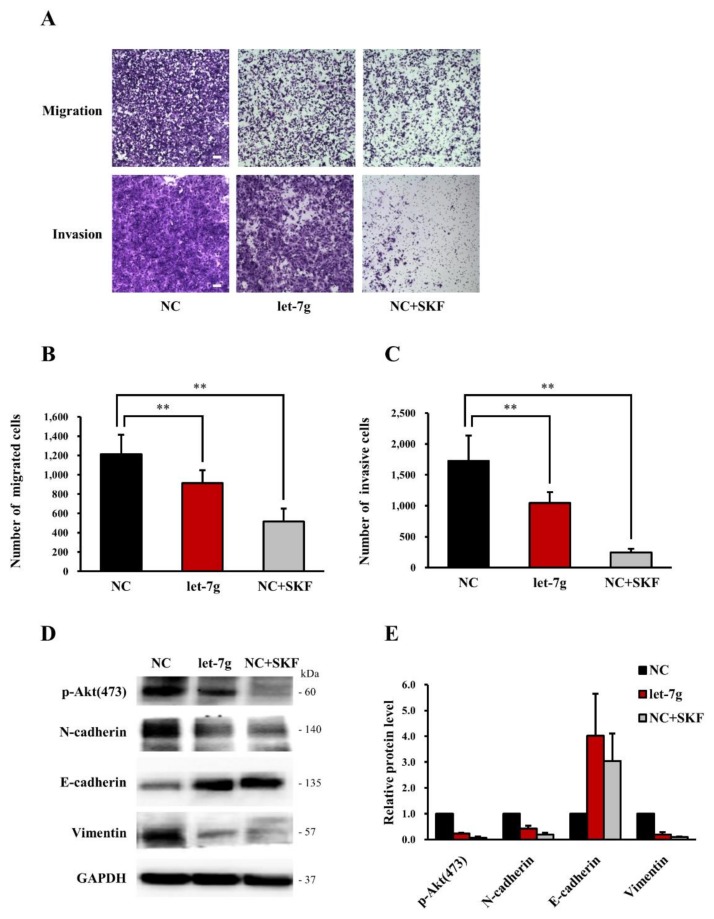
Effect of *let-7g* and a store-operated calcium (SOC) channel inhibitor on the motility of colorectal cancer cells. (**A**) DLD-1 cells transfected with a miRNA negative control and *let-7g* were subjected to transwell migration and invasion assays. Cells were incubated with or without 10 μM SKF96365 for 48 h and then analyzed by imaging. Scale bar: 100 μm. (**B**,**C**) DLD-1 cell migration and invasion were quantified by calculating the number of stained cells. (**D**) Epithelial-mesenchymal transition (EMT)-related protein levels in DLD-1 cells transfected with the miRNA negative control and *let-7g* plus treatment with or without 10 μM SKF96365 were analyzed by Western blotting. The protein expression of glyceraldehyde 3-phosphate dehydrogenase (GAPDH) was assessed as an internal control. (**E**) Western blots of EMT-related protein expressions in DLD-1 cells were quantified. **, statistically significant at a *p*-value of <0.01.

A previous study demonstrated that SOC is critical during CRC progression and metastasis and indicated suppressive effects of SOC inhibitors on CRC migration [26]. To further investigate whether the mechanism by which *let-7g* inhibits CRC migration is correlated with the underlying SOC inhibition, we determined and compared the influences of *let-7g* and SKF96365, a SOC inhibitor, on cell motility. Results revealed that both *let-7g* and SKF96365 inhibited the migration and invasion of DLD-1 cells (Figure 4A). The numbers of migrating and invasive cells significantly decreased with *let-7g* overexpression and SKF96365 treatment (Figure 4B,C). These results suggest that *let-7g* and the SOC inhibitor had similar inhibitory effects on CRC cell migration and invasion. Based on these results, we further examined the influences of *let-7g* and the SOC inhibitor on the epithelial-mesenchymal transition (EMT), which was indicated to be involved in the progression and metastasis of CRC cells [27,28]. Our results showed that both *let-7g* and SKF96365 increased E-cadherin, an epithelial marker and decreased mesenchymal markers including N-cadherin and vimentin in DLD-1 cells, reflecting a reduced migratory ability (Figure 4D,E). Additionally, expression of phospho-Akt was found to be inhibited by *let-7g* and SKF96365, suggesting suppressive influences of *let-7g* and the SOC blocker on tumor progression, including cell proliferation and metastasis (Figure 4D,E). Our findings indicated that both *let-7g* and the SOC inhibitor can block CRC cell motility.

### 2.8. Let-7g Attenuates SOC Influx in Vitro

**Figure 5:**
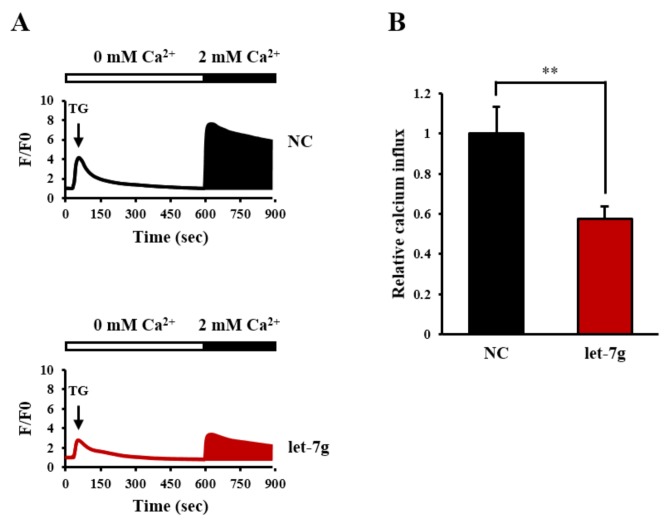
Effect of *let-7g* on store-operated calcium (SOC) entry (SOCE) in colorectal cancer cells. (**A**) The thapsigargin (TG)-induced Ca^2+^ influx via SOC in miRNA negative control and *let-7g*-transfected Caco-2 cells. During calcium imaging, cells were treated with TG followed by 2 mM Ca^2+^ to induce calcium release from the endoplasmic reticulum (ER) and to trigger extracellular influx, respectively. (**B**) Ca^2+^ signals were quantified by calculating the black area beneath the Ca^2+^ curve caused by 2 mM Ca^2+^. **, statistically significant at a *p*-value of <0.01.

Because both *let-7g* and the SOC inhibitor can block CRC cell motility, we further examined the effects of *let-7g* on store-mediated calcium influx. Thapsigargin (TG), an inhibitor of sarco/endoplasmic reticular Ca^2+^ ATPase (SERCA) pumps, was used to trigger classical SOC entry (SOCE). The intracellular calcium imaging system was applied to detect TG-induced calcium release of the ER in a calcium-free environment, following extracellular calcium influx through the SOC channel by the addition of 2 mM calcium. Our data showed that TG-induced SOCE was significantly inhibited by *let-7g* in Caco-2 cells (Figure 5).

### 2.9. Let-7g Inhibits Colorectal Tumor Growth in Vivo

**Figure 6:**
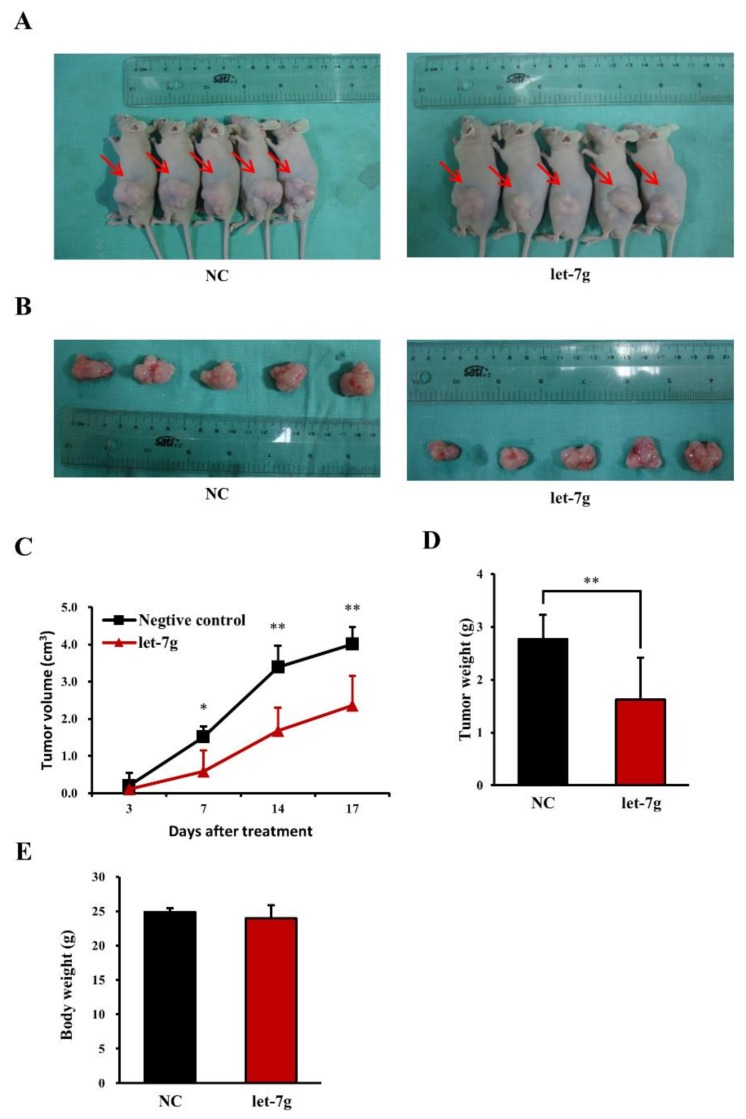
Effect of *let-7g* on colorectal cancer cell growth in nude mice. (**A**) Nude mice orthotopically transplanted with Caco-2 cells were treated with interval injections of a liposome-entrapped miRNA negative control and *let-7g*. The orthotopic tumor size was imaged. (**B**) Orthotopic tumor tissues of nude mice were surgically removed and imaged. (**C**) Orthotopic tumor sizes were measured at 3, 7, 14 and 17 days after tumor implantation. (**D**) The tumor weight of surgically removed tumor tissues were measured. (**E**) The body weights of nude mice were measured. **, statistically significant at a *p*-value of <0.01.

To extend the findings from in vivo cell growth and motility in an animal model, tumor xenograft models were used. In the study, mice were subcutaneously injected with Caco-2 cells to induce tumor formation. Three days later, the mice were treated with either the miRNA negative control or *let-7g* by an intraperitoneal injection of liposomes containing each miRNA twice a week. By continually measuring the tumor volume during 17 days after the initial injection of CRC cells, we observed an inhibitory influence of *let-7g* on CRC growth in mice (Figure 6C). After 17 days, smaller colorectal tumors were also detected in mice treated with *let-7g* compared to those in mice with injection of the miRNA negative control (Figure 6A). We further removed and measured tumor tissues from mice as shown in Figure 6B. Our results revealed that both the tumor volume and weight of *let-7g*-treated mice were significantly lower than those of mice treated with the microRNA negative control (Figure 6C,D). Notably, the body weight of mice in the two groups showed no significant difference, suggesting no adverse effects of *let-7g* on physiological functions of the mice (Figure 6E). Our finding from the in vivo study demonstrated the strong suppressive effect of *let-7g* on CRC growth in a mouse model.

## 3. Discussion

This study combined the TCGA database and a Taiwanese CRC patient cohort study to test the role of *let-7g* in CRC progression. In the TCGA database, a significant correlation between reduced *let-7g* expression and lymphatic invasion was observed. A second cohort validation study in Kaohsiung Medical University Hospital indicated that tissues from CRC patients had decreased levels of *let-7g*. In addition, functional studies revealed that *let-7g* inhibited CRC cell migration and invasion, vimentin expression and store-operated calcium influx. Strikingly, results from the *in vivo* animal studies further supported the protective effects of *let-7g* treatment on CRC progression. Hence, the expression level of *let-7g* can influence physiological changes of CRC through calcium-dependent cell migration pathways.

Decreased expression levels of *let-7g* were reported in many types of cancer, including hepatocellular carcinoma (HCC) [15,18], lung cancer [29], breast cancer [16] and gastric cancer [30]. In HCC, a lower level of *let-7g* was reported to be correlated with metastasis and poor overall survival [15]. Breast cancer patients with lower *let-7g* expression also showed poor overall and recurrence-free survival [16]. Similarly, down-regulation of *let-7g* was reported to correlate with a poor prognosis in gastric cancer patients [30]. Previous studies considered the potential effects of *let-7g* on cancer progression; here, our results from a TCGA data analysis (RNA-sequencing data) confirmed the association between reduced *let-7g* expression and CRC progression. Additionally, a decreased expression of *let-7g* in tumor tissues observed in our CRC cohort supported the crucial role of *let-7g* in CRC development.

Several recent studies have been proposed for a role of *let-7g* in CRC progression. Nakajima et al. reported a significant increase of *let-7g* in tumor tissues from 21 CRC patients [13]. In addition, measuring the serum level of miRNAs indicated a significantly higher expression of *let-7g* in CRC patients than that in healthy individuals [25]. However, another quantitative result revealed no significant difference in *let-7g* expression between tumor and adjacent stromal tissues in 25 rectal cancer patients [31]. It seems that the evidence cannot be reconciled with each other. However, in light of the fact that there are so many examples that indicated store-operated calcium influx is a critical pathway in cancer cell migration and proliferation [32,33,34], the functional results in the current study indicate the inhibitory effects of *let-7g* in store-operated calcium entry. Consistent with this concept, we observed low expression of *let-7g* in CRC tissues. Most studies addressing the roles of *let-7g* are plagued with a number of uncertainties. We attribute this to the small clinical sample size, which may lack of statistical power in the analysis. Further carefully designed studies are needed in order to evaluate the influence of *let-7g* in carcinogenesis.

How *let-7g* results in CRC migration through calcium signals is unclear at present. Nevertheless, results showed that *let-7g* can at least target, through attenuation of SOC signals, EMT pathways, which involve the motility of cancer cells. Consistent with our findings, inhibitory functions of *let-7g* on cell cancer cell motility were addressed. Overexpression of *let-7g* has been demonstrated to reduce the proliferation, migration and invasion of HCC cells [35]. By inhibiting *let-7g*, breast cancer cell migration and invasion were promoted via the downregulation of Grb2-associated binding protein 2 (GAB2) and fibronectin 1 (FN1) expressions that led to subsequent activation of MAPK pathways [16]. Indeed, our findings can be nicely incorporated into the model proposed by Qian and colleagues. *let-7g* reduces the motility of cancer cells such that basal SOC might be insufficient to support FN1 expression and MAPK pathway activation.

Dysregulated SOC pathways were widely reported to promote cancer cell migration, invasion and metastasis in various cancers [32,33,34]. Recent findings demonstrated that stromal interaction molecule 1 (STIM1), an ER calcium sensor of SOCE, promoted the motility of CRC cells [26]. Notably, STIM1 can be downregulated by miRNA-195 and miRNA-185, which results in abrogation of CRC cell migration and invasion [26,36,37]. In our study, we identified the functional role of *let-7g* in SOC mobilization. The mechanisms by which *let-7g* inhibited SOC signaling are as yet unclear. Previous studies reported several genes (*HMGA2*, *K-Ras*, *Bcl-xL*, *COL1A2*, *GAB2* and *FN1*) as potential targets of *let-7g*. Although various genes were proposed to function in cancer cell motility pathways, there is, as yet, no widely accepted candidate for *let-7g* in CRC cells. Additionally, it is not clear whether *let-7g*’s mediation of calcium signals contributes equally to the activation of all of these genes or whether one gene is preferentially involved [15,16,38,39,40].

## 4. Materials and Methods

### 4.1. Data Acquisition and Patient Categorization of the TCGA CRC Cohort

We downloaded CRC (including colon adenocarcinoma (COAD) and rectum adenocarcinoma (READ)) patients’ primary tumor data with available miRNA sequencing and RNA sequencing (version 2) information using the TCGA Assembler [41]. The queried sequencing data were further processed using the ProcessmiRNASeqData and ProcessRNASeqData functions as implemented in the TCGA Assembler. We next extracted *let-7g* expression data (RPM), which were log-transformed, and then queried the corresponding clinical profiles of CRC patients. The RNA sequencing expression data of these patients were processed as follows: We considered a gene to be expressed if it was present at a level of >0 in 80% of the samples, resulting in 16,387 transcripts. We further defined outliers as those samples which were more than five standard deviations above the mean connectivity based on a signed, weighted bi-weight mid-correlation using the WGCNA package and removed one outlier sample, resulting in 327 samples [42]. Finally, expression values were normalized (rounding cutoff equal to 1) using the sRAP package.

We categorized CRC samples based on *let-7g* expression values. In this study, we applied two criteria (20% or 25%) to perform patient categorization. We defined CRC patients with a *let-7g* expression value of larger than the upper quartile or quintile as the *let-7g* overexpression group; and CRC patients with a *let-7g* expression value smaller than the lower quartile or quintile as the *let-7g* low-expression group. Based on these criteria, 83 (upper quartile) and 82 (lower quartile) or 67 (upper quintile) and 66 (lower quintile) samples were selected for downstream analysis.

### 4.2. Analysis of Clinical Demographics in TCGA CRC Samples

The 165 CRC patients selected based on quartile categorization were included to assess the correlation between the *let-7g* expression status (*let-7g* high-expression or low-expression) and patients’ clinical profiles (including gender, diagnosed age, depth of invasion, lymph node metastasis, distant metastasis, UICC staging, vascular invasion, lymphatic invasion and perineural invasion). A logistic regression model under a binomial distribution was fitted for the association test. We also conducted the same statistical model by adjusting for the effects of gender, age and tumor location (colon or rectum). A *p*-value of <0.05 was considered statistically significant.

### 4.3. Analysis of Transcript Expression Profiles Based on Sample Categorization

Differentially expressed genes (DEGs) were identified by comparing *let-7g* high-expression and low-expression based on the quartile or quintile categorization. We applied a moderated *t*-test to identify DEGs [43]. We further defined significant DEGs as genes having a Benjamini and Hochberg (BH)-adjusted *p*-value of <0.05. To visualize expression patterns of identified DEGs in CRC samples, the top 100, 200 and 300 DEGs were extracted for a heatmap analysis. In addition, unsupervised agglomerative hierarchical clustering was performed on these most variant DEGs with the following parameters: distfun = “euclidean” and hclustfun = “average”. We further conducted a non-negative matrix factorization (NMF) analysis using the NMF package [44]. In the NMF analysis, the stability of consensus matrices under two to six factorization ranks was iteratively conducted using the Brunet algorithm [45]. To evaluate the reproducibility of the clustering effect, the top 100, 200 and 300 DEGs under quartile and quintile categorization were all separately analyzed. Furthermore, cluster signatures were extracted from the output results of the NMF analysis and further correlated with patients’ clinical features.

### 4.4. DIANA-Annotated Targets Gene Set Enrichment Analysis

Target genes of *let-7g* were queried based on human orthologues using the online DIANA TOOLS TarBase v7.0, an experimentally supported and manually curated miRNA target database [46]. A gene set analysis was performed using a permutation test to compare *p*-values and multiples of change of 1655 *let-7g* targets and the remaining genes. The *p*-values were log_10_-transformed and the absolute value of the multiple of changes underwent a log_10_ transformation so that larger values represented higher correlations (for the *p*-value) or larger changes (for multiples of change) across the *let-7g* overexpression and low-expression groups. We generated a null distribution by 10,000 random permutations of the gene labels and calculated the gene set enrichment score (ES) based on the Kolmogorov–Smirnov statistic. Then, we scaled the ES by subtracting the mean and dividing by the standard deviation of the permutated ES to obtain a normalized ES (NES; i.e., the Z-score). Finally, significant *p*-values were calculated from the resulting Z-scores.

### 4.5. Gene Ontology (GO) Enrichment and Pathway Analysis of DEGs in CRC Samples

Following DEG identification, gene set analysis were performed. We first extracted a smaller set of DEGs as the most important features for the metagene, which were generated from the clustering of most variant DEGs based on NMF analysis and corresponded to cluster signatures as *let-7g*-associated CRC subpopulations, by using a default feature extraction function implemented in the NMF package [44,47]. Based on the result of feature extraction, parts of features corresponding to specific cluster signatures were further selected for capturing *let-7g*-associated biological features. We conducted a GO biological process (BP) term, and Kyoto Encyclopedia of Genes and Genomes (KEGG) over-representation analysis (ORA) was carried out using the GOstats package to identify enriched BP terms and KEGG pathways [48]. We selected a *p*-value cutoff of 0.05 in the ORA. We also carried out signaling pathway impact analysis (SPIA) using the SPIA package to identify aberrant KEGG pathways between the *let-7g* overexpression and low-expression groups [49]. Perturbation *p*-values were calculated using 20,000 bootstrap iterations. Finally, the pathways that had *p*-values of <2 × 10^−6^ were considered as significantly perturbed. The KEGG pathways were visualized using the Pathview package [50].

### 4.6. Patient Recruitment and Sample Preparation

In total, 20 patients diagnosed with CRC were enrolled at Kaohsiung Medical University Hospital. The study was conducted with approval from the institutional review board of Kaohsiung Medical University, Taiwan (KMUHIRB-2012-03-02 (II)). All patients were provided with informed consent for the study prior to data and sample collection. Both tumor and adjacent normal tissues were surgically collected from CRC patients and homogenized in TRIzol reagent (Thermo Fisher Scientific, Waltham, MA, USA). Total RNAs were then extracted from homogeneous tissue samples following the manufacturer’s instructions and applied to a polymerase chain reaction (PCR) for a gene expression analysis.

### 4.7. Reverse-Transcription Quantitative Polymerase Chain Reaction (RT-qPCR)

An RT-qPCR was performed to synthesize complementary (c)DNA from 1 μg of isolated RNA using an RT kit (Thermo Fisher Scientific). Following the cDNA synthesis, *let-7g* and *miR191* expressions were determined by a quantitative (q)PCR method using a TaqMan Gene Expression Assay (Thermo Fisher Scientific) performed on a StepOnePlus Real-Time PCR System plus StepOne software version 2.2.2 (Thermo Fisher Scientific). The relative gene expression level was calculated from the ratio of the *let-7g* cycle threshold (C_T_) to the *miR191* C_T_ value.

### 4.8. Cell Culture and Transfection

Caco-2 and DLD-1 cells, obtained from American Type Culture Collection (ATCC, Manassas, VA, USA), were grown in Dulbecco’s modified Eagle’s medium (DMEM) with 10% fetal bovine serum (FBS) and 1% penicillin at 37 °C in 5% CO_2_. Before conducting the following experiments, cells were transfected with either a miRNA negative control (100 nM) or *let-7g* mimic (100 nM) (Thermo Fisher Scientific) using Lipofectamine (Thermo Fisher Scientific) in Opti-MEM (minimum essential medium; Thermo Fisher Scientific) for a least 16 h. Transfected cells were suspended using 0.05% trypsin (Thermo Fisher Scientific) in phosphate-buffered saline (PBS) and used for the following experiments.

### 4.9. Cell Migration by a Wound-Healing Assay

Cells were seeded into culture inserts (ibidi, Munich, Germany), which were placed in 6-well plates. Each well contained 5 × 10^4^ cells in one insert supplied with DMEM at 37 °C in 5% CO_2_. Following incubation overnight, the culture inserts were removed, and cells were then treated with or without 25 ng/mL EGF in serum-free DMEM for 24 h. Cell migration was analyzed by measuring the wound healing of Caco-2 and DLD-1 cells using microscopic imaging systems (Leica Camera, Wetzlar, Germany) and Luma-controller (Etaluma, Carlsbad, CA, USA), respectively.

### 4.10. Cell Migration by a Biosensor Approach

An x’Celligence Biosensor System based on a real-time cell analyzer (RTCA) dual plate (DP) instrument (Roche Diagnostics, Mannheim, Germany) was used to monitor and evaluate cell migration according to the manufacturer’s instructions. The cell invasion and migration (CIM)-plate was monitored once every 15 min. Data were collected and analyzed using RTCA v1.2 software (Roche Diagnostics).

### 4.11. Transwell Cell Migration and Invasion Assays

Cells were suspended in 500 μL of serum-free DMEM and seeded into BD Falcon culture inserts (BD Biosciences, San Jose, CA, USA) for the cell migration assay or into BD BioCoat™ Matrigel Invasion Chambers (BD Biosciences) for the cell-invasion assay. Each insert or chamber contained 10^5^ cells and was placed in 24-well plates filled with 1 mL of DMEM with 10% FBS. After a 48-h incubation, non-migrating cells that remained on the upper surface of the membrane in the insert of the chamber were removed by scrubbing. Migrated cells on the reverse side of the membrane were fixed in 100% methanol and then stained with 0.1% crystal violet. Cell migration and invasion were evaluated by measuring the number of migrated cells using microscopic imaging (Leica Camera).

### 4.12. Western Blotting

Cells were harvested and lysed in radio-immunoprecipitation assay (RIPA) buffer with a protease inhibitor, then total cell lysates were extracted to perform a Western blot analysis. Total cellular proteins were first separated by 12.5% sodium dodecylsulfate polyacrylamide gel electrophoresis (SDS-PAGE) and then transferred onto polyvinyl difluoride (PVDF) membranes (GE Healthcare, Little Chalfont, Buckinghamshire, UK). Membranes were blocked in 5% bovine serum albumin (BSA) in 0.1% PBS/Tween-20 (PBST) for 1 h and then incubated with a primary antibody against p-Akt (Cell Signaling Technology, Danvers, MA, USA), N-cadherin (Cell Signaling Technology), E-cadherin (Cell Signaling Technology), vimentin (Santa Cruz Biotechnology, Santa Cruz, CA, USA), or GAPDH (Santa Cruz Biotechnology) followed by incubation with a horseradish peroxidase (HRP)-conjugated secondary antibody (GeneTex, Irvine, CA, USA). The signal of the target proteins was enhanced by a chemiluminescent HRP substrate (EMD Millipore, Billerica, MA, USA) and then detected by an electrochemiluminescence (ECL) detection system.

### 4.13. Calcium Imaging

Cells were seeded onto 20-mm coverslips in 6-well plates and grown in DMEM at 37 °C in 5% CO_2_. After incubation overnight, cells were incubated with 1 μM Fluo-4-AM (Molecular Probes, Eugene, OR, USA) in calcium-free buffer at 37 °C for 20 min. Following Fluo-4-AM staining, cells were treated with 2 μM thapsigargin (TG) and then a 2 mM calcium solution to trigger calcium release by the endoplasmic reticulum (ER) and extracellular calcium influx, respectively. Real-time intracellular Ca^2+^ signals were detected using an MT 20 illumination system (Olympus, Melville, NY, USA) mounted on an Olympus Cell^R IX81 fluorescence microscope with a UPLanApo 10× objective lens and estimated based on the fluorescent intensities emitted upon excitation of consecutive 488-nm pulses of light at a resolution of 1376 × 1038 pixels.

### 4.14. Animal Study

In this study, male Nu/Nu mice (5 weeks old with an average weight of 20.3 g; from National Laboratory Animal Center, Taiwan) were subcutaneously injected with 1 × 10^7^ number of Caco-2 cells. All the mice were injected intraperitoneally with the liposome containing microRNA negative control or let-7g twice a week. And tumor volumes were calculated using the equation (*L* × *W*^2^)/2, where *L* and *W* are the larger and smaller tumor dimensions, respectively. After 17 days, the mice were sacrificed, and all the tumors were excised and weighed. The protocol of animal experiments in this study was reviewed and approved by the Institutional Animal Care and Use Committees (IACUCs) of Kaohsiung Medical University (approval no: 106083) and Taipei Medical University (approval no: LAC-2018-0346 and LAC-2019-0052).

### 4.15. Statistical Analysis

We performed all statistical and bioinformatics analysis using R software (http://www.r-project.org/; http://cran.r-project.org/) and Bioconductor (http://www.bioconductor.org/). The statistical difference in *let-7g* expression between tumor and normal tissues in CRC patients was determined by the Wilcoxon test. A statistical analysis to compare other experimental results was performed with Student’s *t*-test. A *p*-value of <0.05 was considered statistically significant and was denoted by *, and a *p*-value of <0.01 was denoted by **.

## 5. Conclusions

In short, our results offer new insights into the clinical consequences of *let-7g* in CRC patients. First, we confirmed the significant association between *let-7g* expression and the clinical status of CRC patients from two independent cohorts. Second, we demonstrated a protective role of *let-7g* in CRC cells via inhibition of cancer cell motility as well as attenuation of SOC signals. Third, our results established the importance of *let-7g* treatment in determining subsequent cancer developmental events in an in vivo model. Finally, our results identified *let-7g* as a rational diagnostic target aimed at CRC metastasis.

## Figures and Tables

**Table 1:** Correlations between clinic pathological features and non-negative matrix factorization (NMF) clusters (quantile categorization) for colorectal cancer patients.

Characteristics	Total Cases	NMF Clusters			*p*-Value	Covariates Adjusted *p* ^a^	FDR Adjusted *p* ^b^
	*n*	Cluster 1 *n* (%)	Cluster 2 *n* (%)	Cluster 3 *n* (%)			
Depth of Invasion							
T1 + T2	28	2 (7.1)	16 (57.1)	10 (35.8)	0.015 *	0.019 *	0.038 *
T3 + T4	136	38 (27.9)	45 (33.1)	53 (39.0)			
Lymphatic Invasion							
No	92	17 (18.5)	37 (40.2)	38 (41.3)	0.026 *	0.044 *	0.044 *
Yes	49	17 (34.7)	13 (26.5)	19 (38.8)			

^a^ Adjusted the effects of diagnosed age, gender and location (colon and rectum); ^b^ Adjusted the effects of diagnosed age, gender and location (colon and rectum), followed by Benjamini-Hochberg multiple correction; * A *p*-value of <0.05 was considered statistically significant. FDR: false discovery rate.

## Supplementary Materials

The following are available online at https://www.mdpi.com/2072-6694/11/4/489/s1, Figure S1: Heatmap of differentially expressed genes (DEGs), Figure S2: Non-negative matrix factorization results of the top 100, 200 and 300 most variant differentially expressed genes (DEGs) based on quartile categorization, Figure S3: Non-negative matrix factorization result of the top 100, 200 and 300 most variant differentially expressed genes (DEGs) based on quintile categorization, Figure S4: Target set enrichment analysis of *p*-values and multiples of change based on quartile (yellow) and quintile (red) categorization, Figure S5: Focal adhesion pathway based on the KEGG graph, Figure S6: Calcium signaling pathway based on the KEGG graph, Figure S7: Extracellular matrix (ECM)-receptor interaction pathway based on the KEGG graph, Figure S8: Cytokine-cytokine receptor interaction pathway based on the KEGG graph, Figure S9: Chemokine signaling pathway based on the KEGG graph, Figure S10: Pathways in cancer based on the KEGG graph, Table S1: Correlations between clinic pathological features and let-7g miRNA expression for 165 TCGA CRC patients, Table S2: Over-representation analysis results of gene ontology (GO) biological process (BP) terms based on 18 NMF cluster-related genes, Table S3: Signaling pathway impact analysis (SPIA) results in CRC patients, Table S4: Correlations between clinic pathological features and *let-7g* expression ratios for 20 postoperative Taiwanese colorectal cancer patients
