# Supplementary material for: Functional Effects of let-7g Expression in Colon Cancer Metastasis
Source: Cancers (Basel). 2019 Apr 6;11(4):489. doi: 10.3390/cancers11040489 (PMC6521310; doi:10.3390/cancers11040489)
Supplement: Supplementary file 1 [file cancers-11-00489-s001.pdf]

Supplementary Material: Functional Effects of *let-7g* Expression in Colon Cancer Metastasis

Che-Mai Chang, Henry Sung-Ching Wong, Chien-Yu Huang, Wen-Li Hsu, Zhi-Feng Maio, Siou-Jin Chiu, Yao-Ting Tsai, Ben-Kuen Chen, Yu-Jui Yvonne Wan, Jaw-Yuan Wang and Wei-Chiao Chang

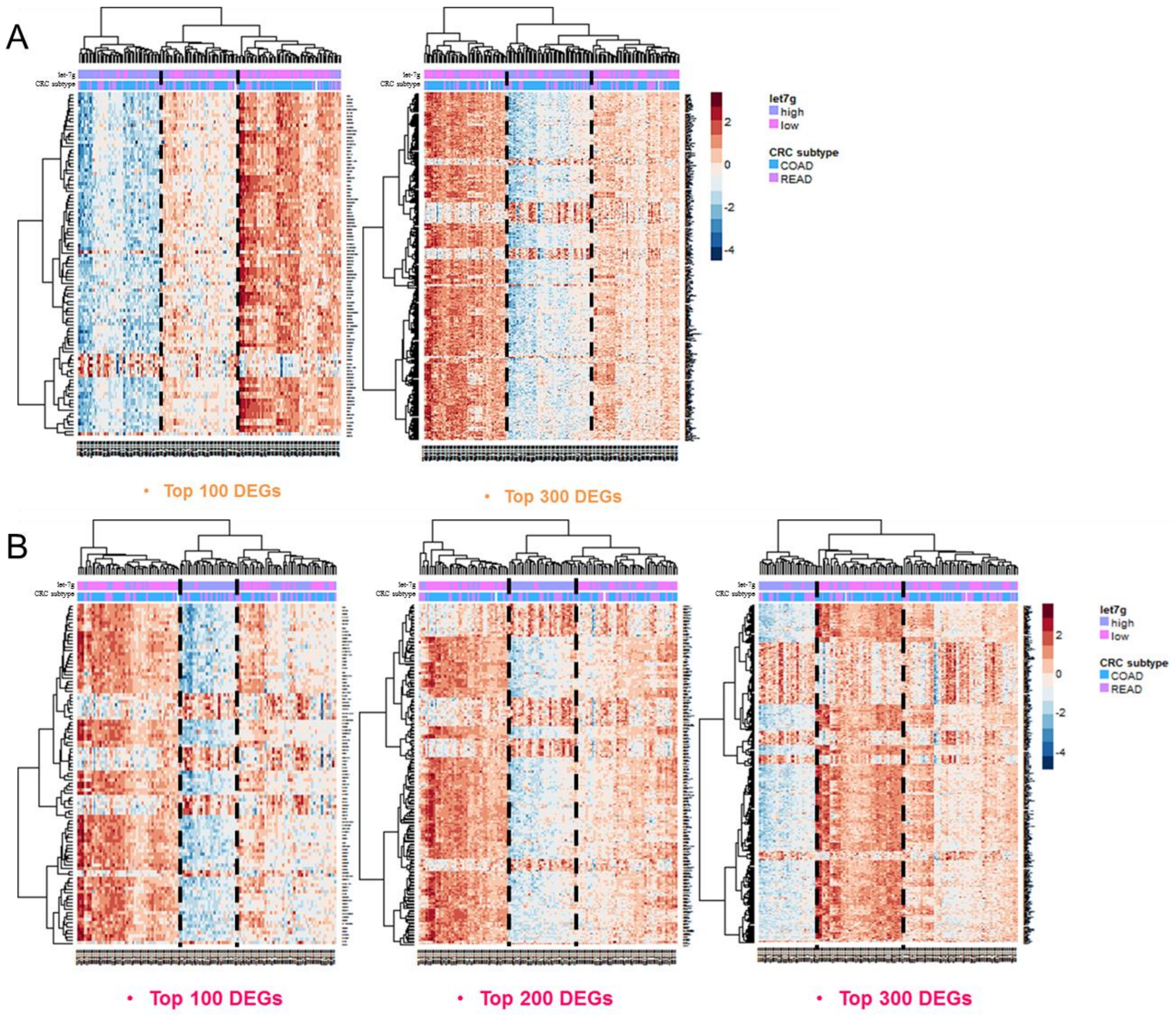

Figure S1. Heatmap of differentially expressed genes (DEGs). (**A**) Heatmaps of the top 100 and 300 most variant DEGs based on quartile categorization of the RNA sequencing profile. Each column represents colorectal cancer (CRC) samples and each row represents genes. Two-way hierarchical clustering was performed, and information of the *let-7g* expression status and the CRC location were mapped. (**B**) Heatmaps of the top 100, 200 and 300 most variant DEGs based on quintile categorization in the RNA sequencing profile. Each column represents CRC samples, and each row represents genes. Two-way hierarchical clustering was performed, and information of the *let-7g* expression status and CRC location were mapped.

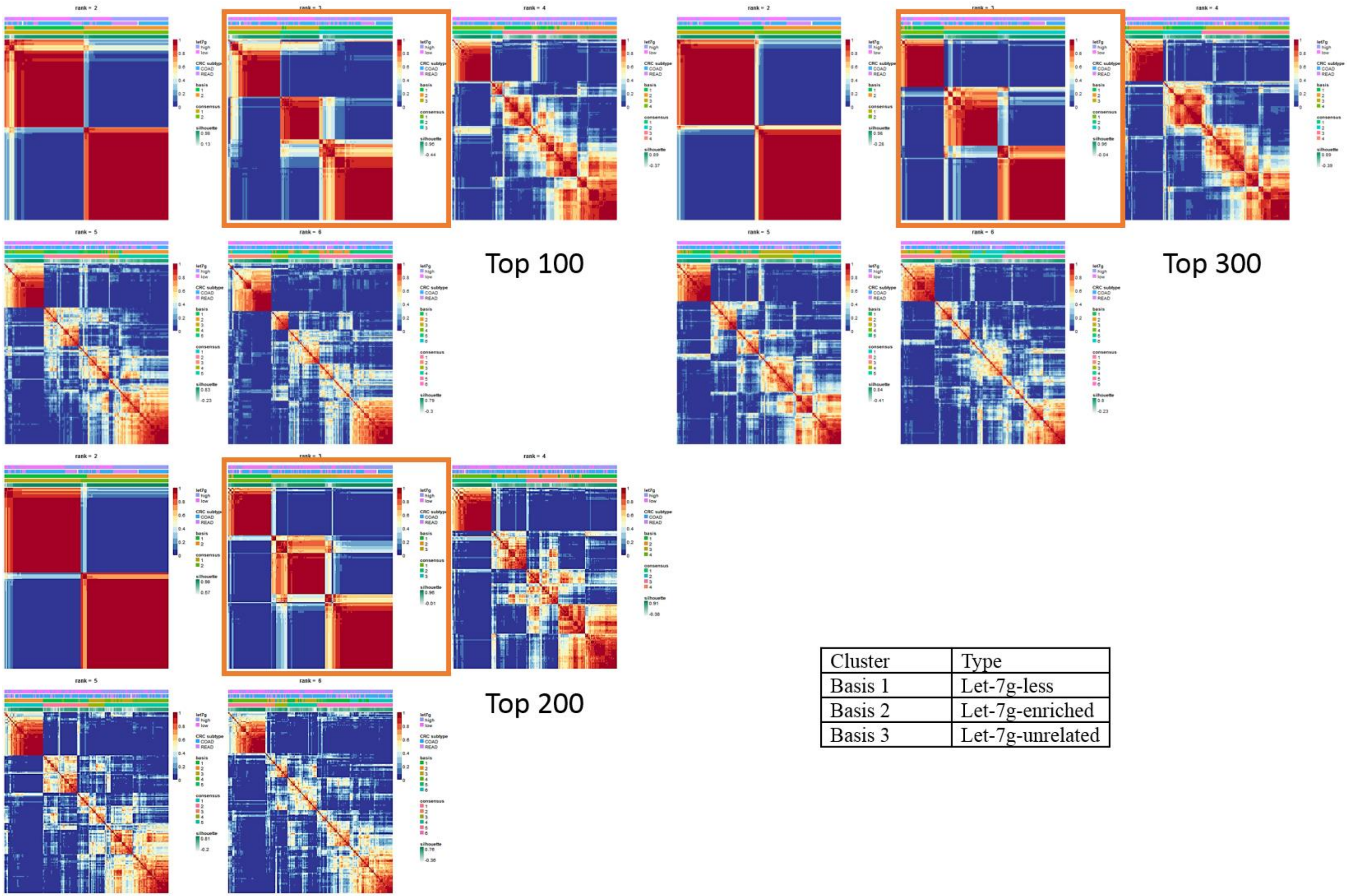

Cluster	Type
Basis 1	Let-7g-less
Basis 2	Let-7g-enriched
Basis 3	Let-7g-unrelated

Figure 2. Non-negative matrix factorization results of the top 100, 200 and 300 most variant differentially expressed genes (DEGs) based on quartile categorization. At each factorization rank (2 to 6), a consensus matrix was plotted by averaging 200 connectivity matrices. The strong consensus for a rank number equal to two indicates a repeatable partitioning of samples into two classes.

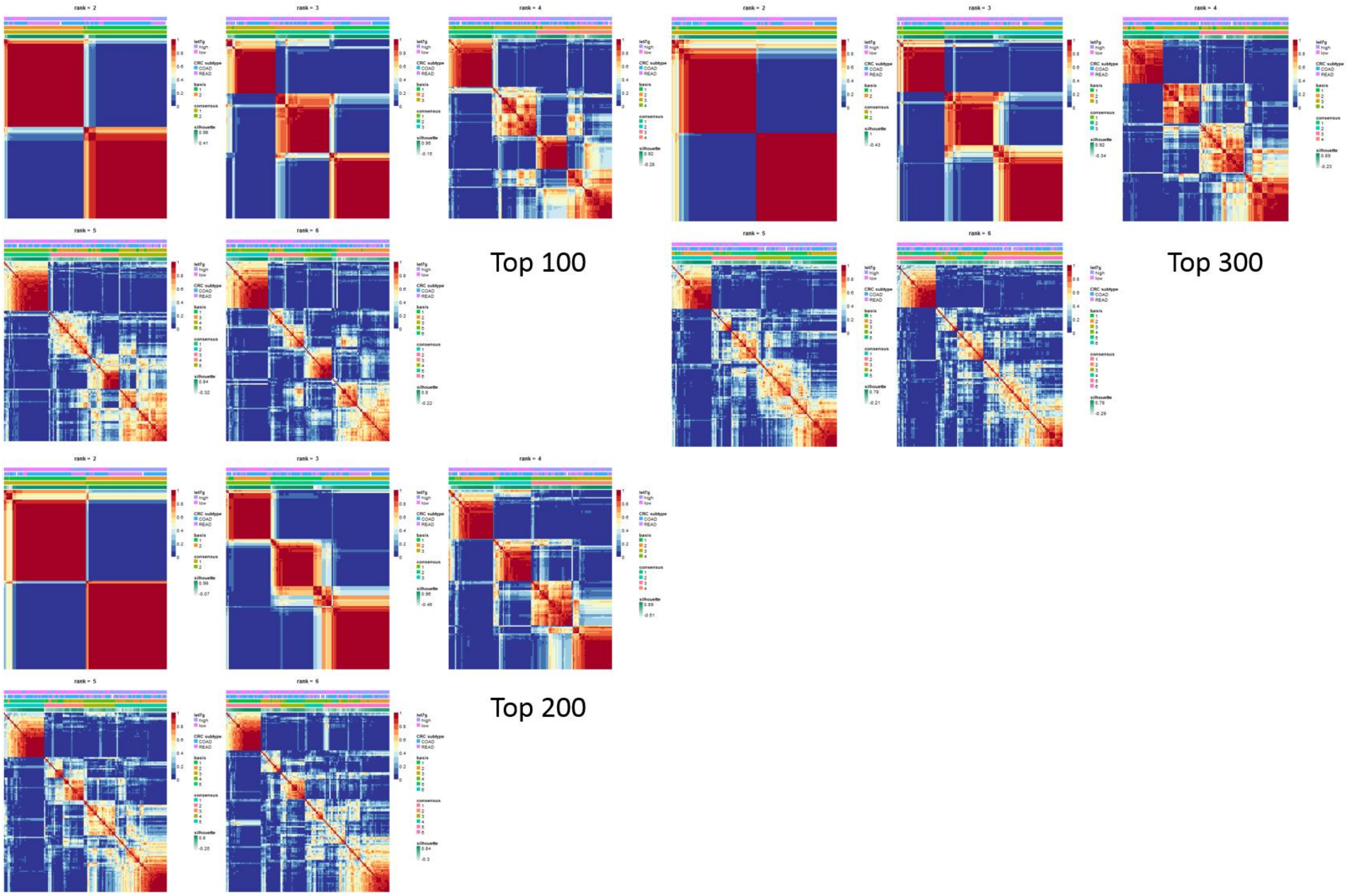

Figure S3. Non-negative matrix factorization result of the top 100, 200, and 300 most variant differentially expressed genes (DEGs) based on quintile categorization. At each factorization rank (2 to 6), a consensus matrix was plotted by averaging 200 connectivity matrices. The strong consensus for a rank number equal to two indicates a repeatable partitioning of samples into two classes.

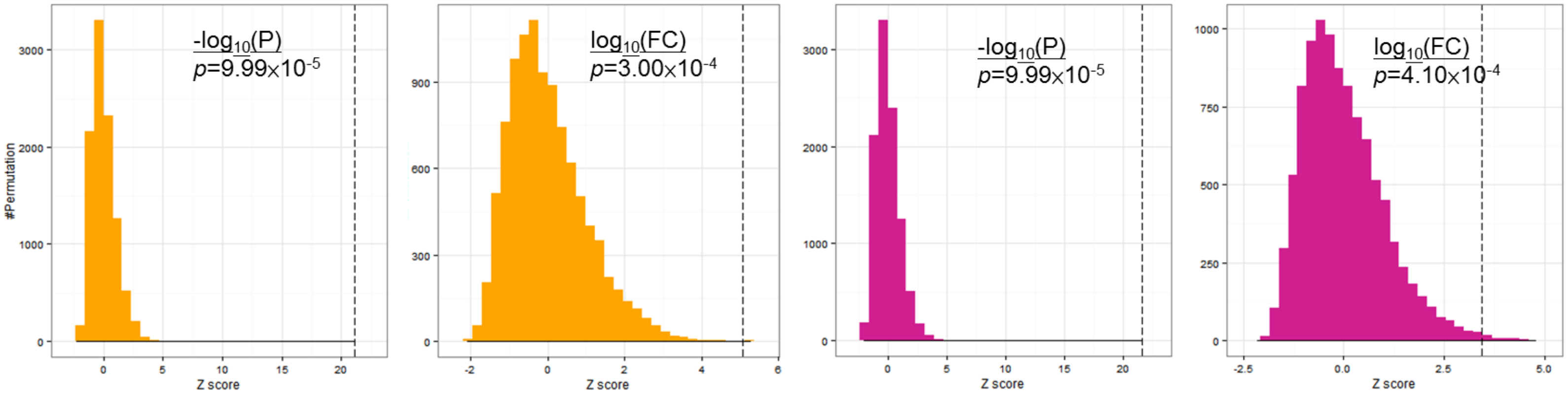

Figure S4. Target set enrichment analysis of *p*-values and multiples of change based on quartile (yellow) and quintile (red) categorization. The null distribution was generated from 10,000 random permutations, and the Z-scores for the targets in each condition are depicted by the dotted line.

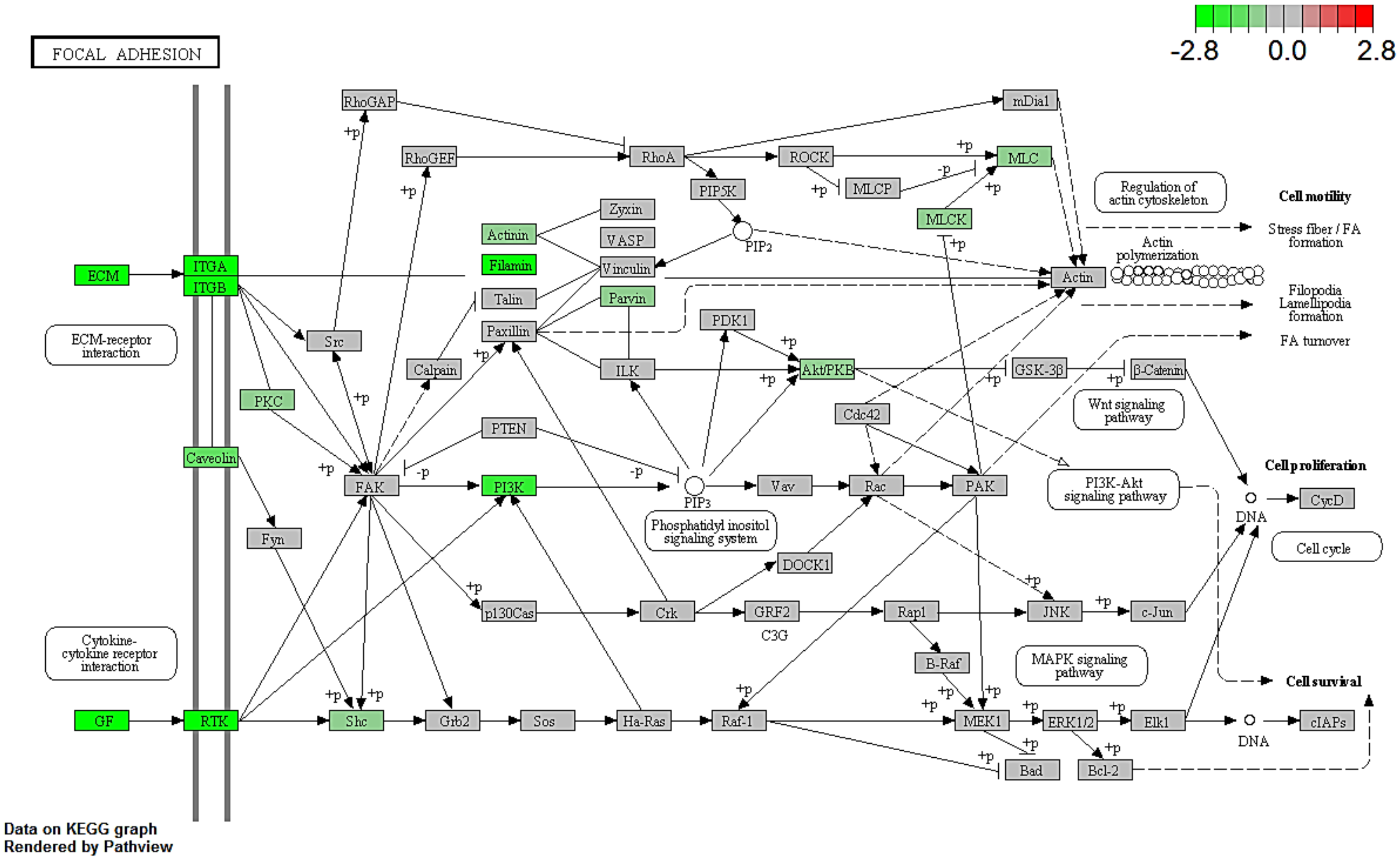

Figure 5. Focal adhesion pathway based on the KEGG graph. Pathway-related genes were mapped by red, grey, or green colors based on the direction and magnitude of the corresponding multiple of change.

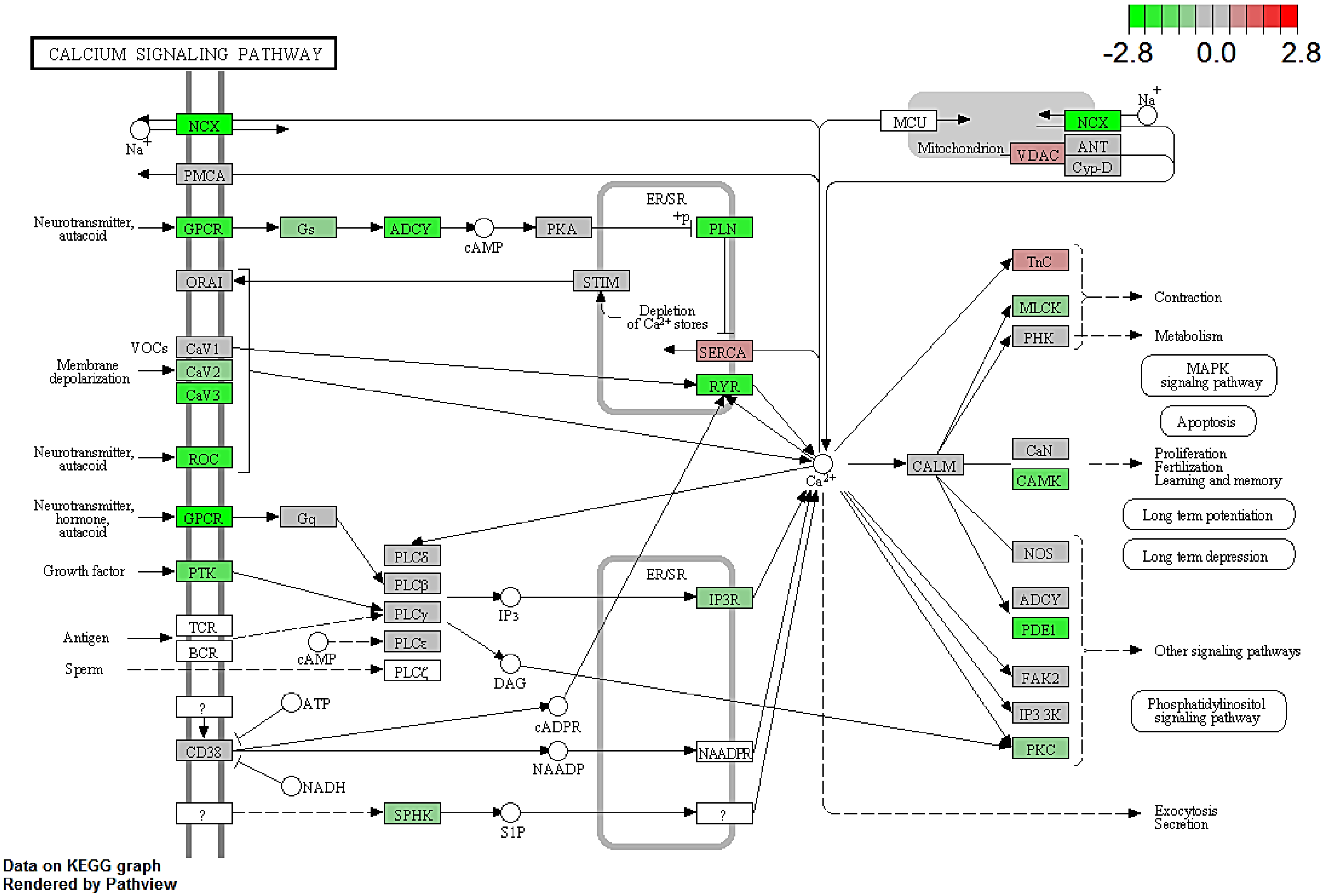

Figure 6. Calcium signaling pathway based on the KEGG graph. Pathway-related genes were mapped by red, grey, or green colors based on the direction and magnitude of the corresponding multiple of change.

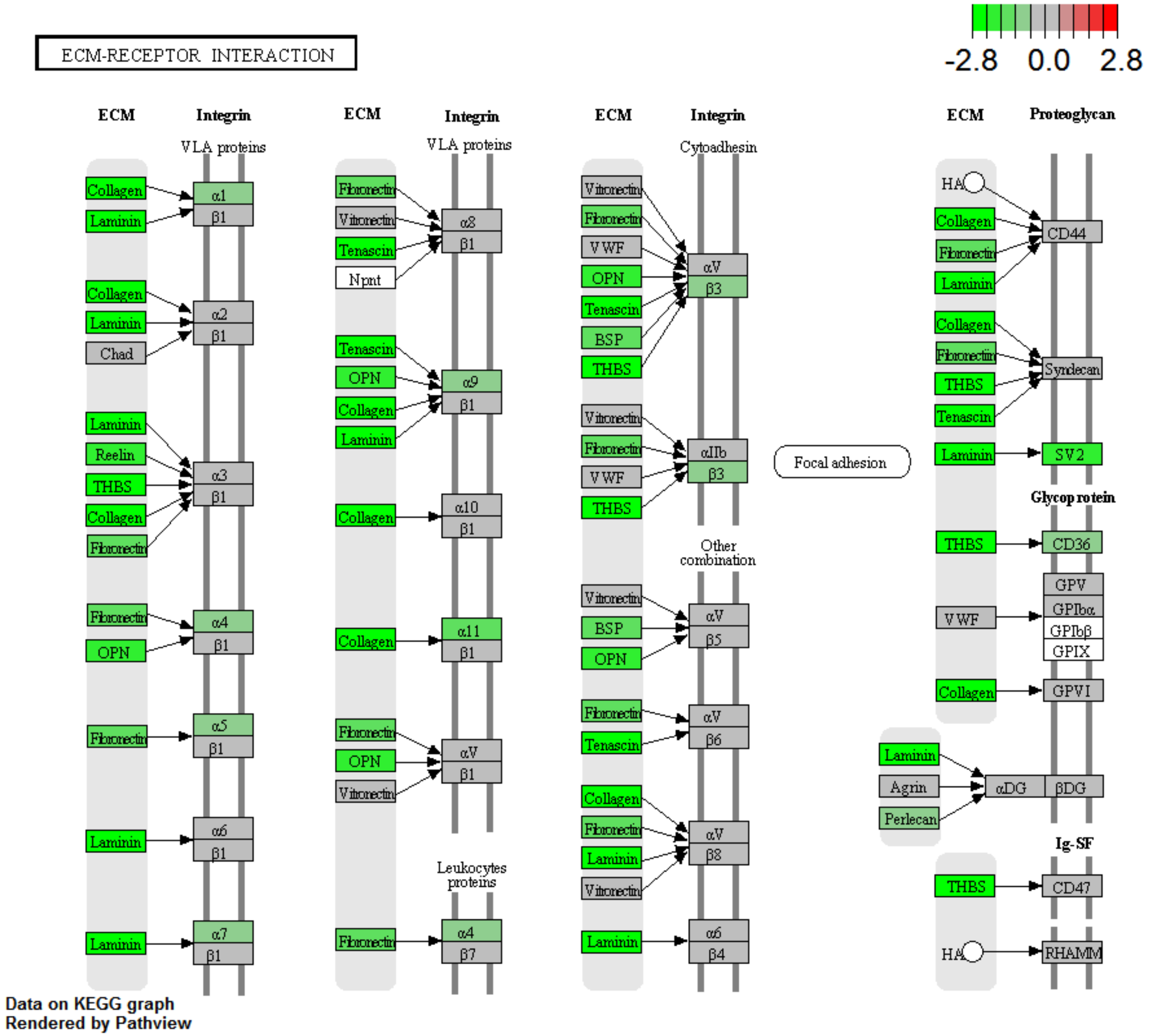

Figure 7. Extracellular matrix (ECM)-receptor interaction pathway based on the KEGG graph. Pathway-related genes were mapped by red, grey, or green colors based on the direction and magnitude of the corresponding multiples of change.

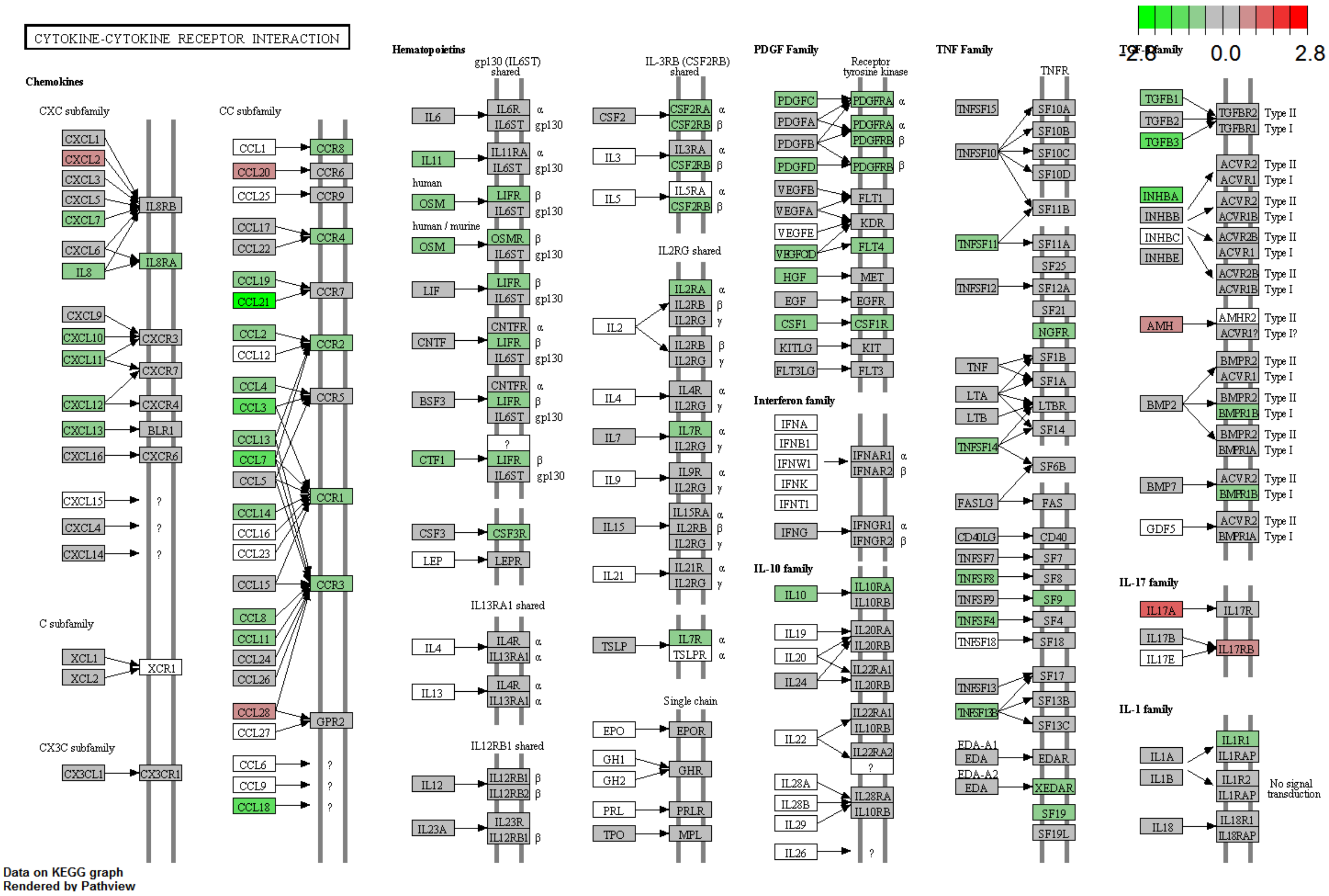

Figure 8. Cytokine–cytokine receptor interaction pathway based on the KEGG graph. Pathway-related genes were mapped by red, grey, or green colors based on the direction and magnitude of the corresponding multiples of change.

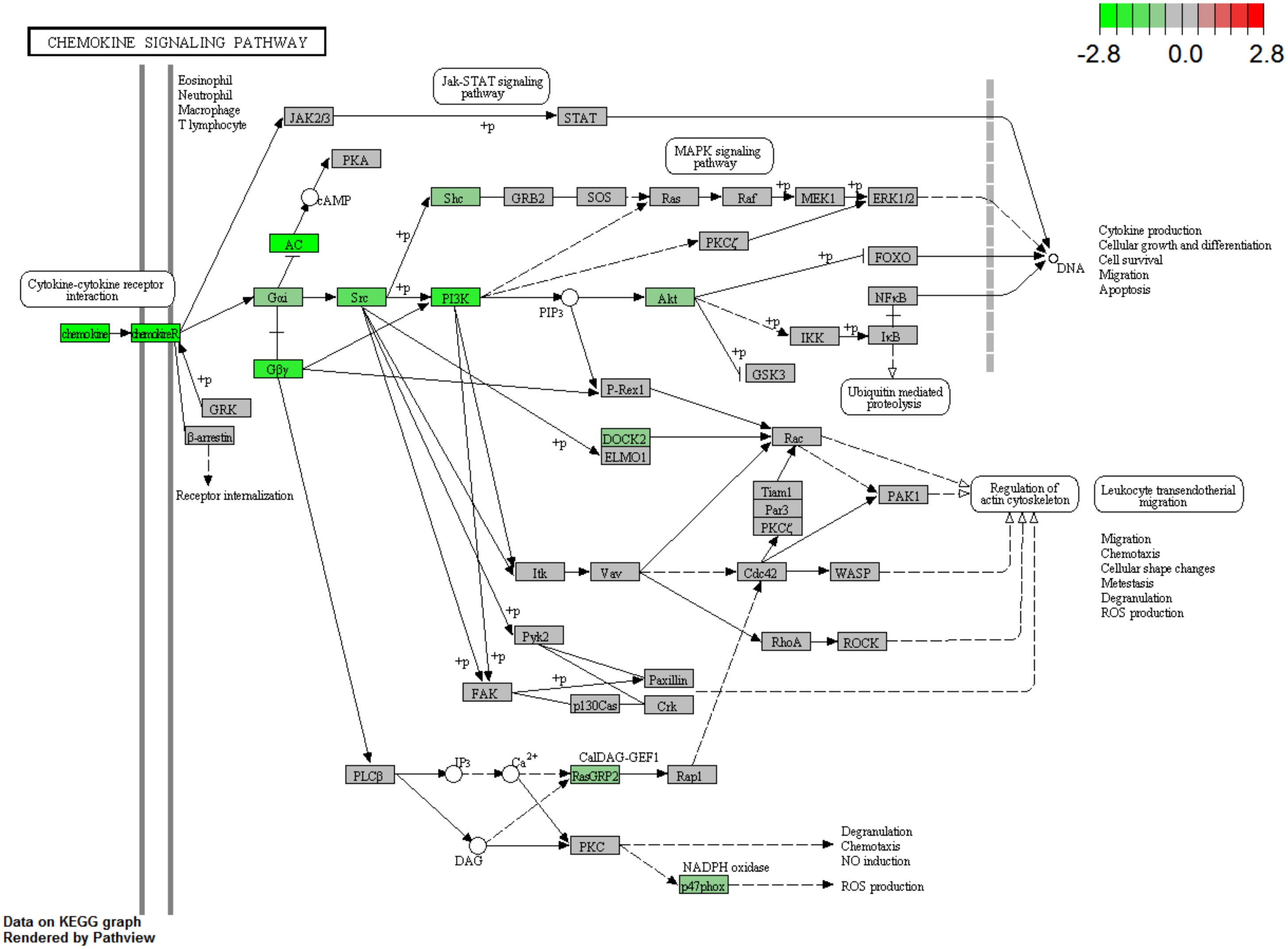

Figure 9. Chemokine signaling pathway based on the KEGG graph. Pathway-related genes were mapped by red, grey, or green colors based on the direction and magnitude of the corresponding multiples of change.

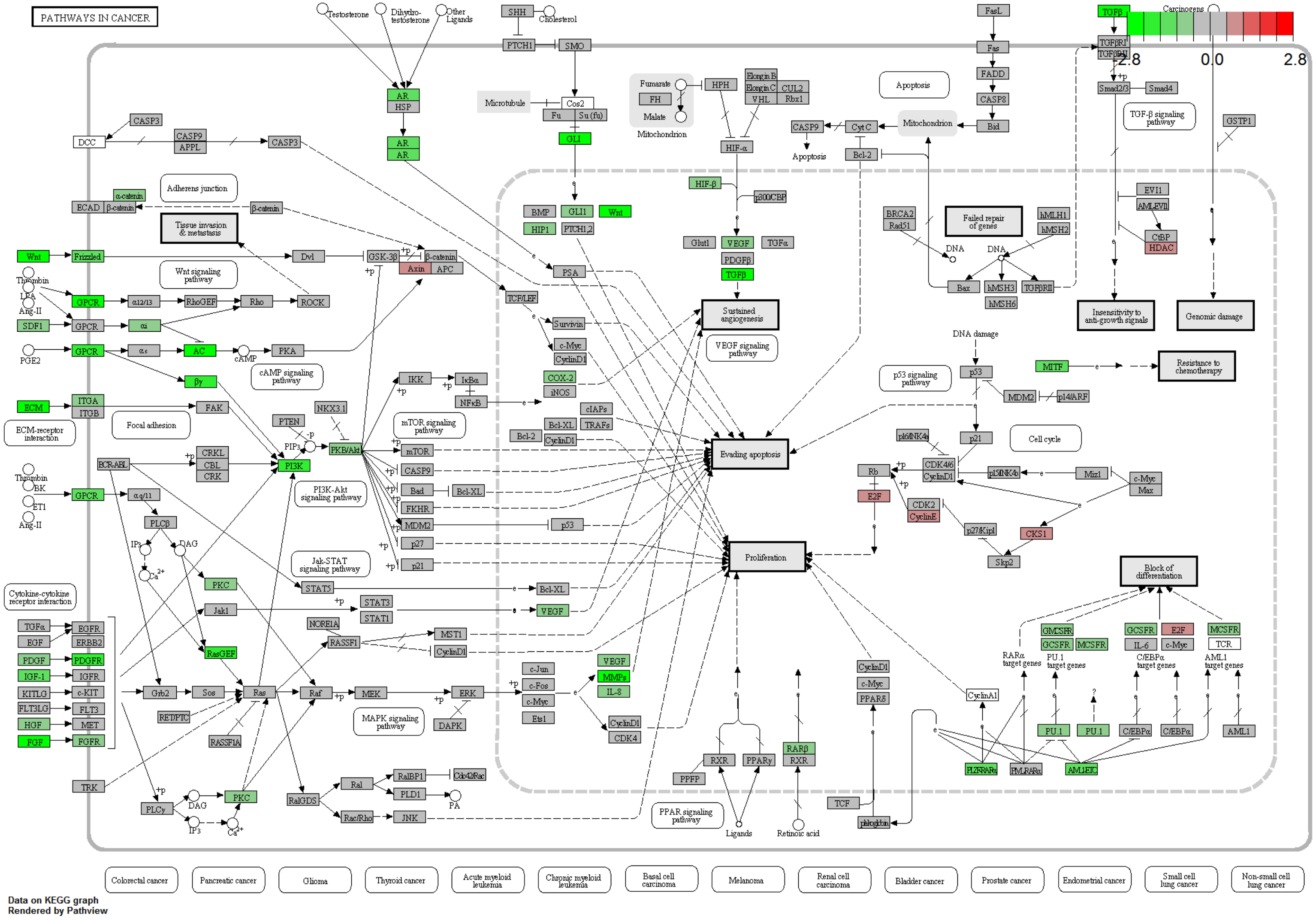

Figure 10. Pathways in cancer based on the KEGG graph. Pathway-related genes were mapped by red, grey, or green colors based on the direction and magnitude of the corresponding multiples of change.

Table S1. Correlations between clinic pathological features and *let-7g* miRNA expression for 165 TCGA CRC patients.

Characteristics	Total Cases	*Let-7g* Expression		*p*-Value	Covariates Adjusted p [a]	FDR Adjusted p [b]
	N	Low N (%)	High N (%)			
Gender						
Female	70	35 (50.0)	35 (50.0)	0.947		
Male	95	47 (49.5)	48 (50.5)			
Diagnosed Age (years)						
<median	85	48 (56.5)	37 (43.5)	0.074		
>median	80	34 (42.5)	46 (57.5)			
Depth of invasion						
T1 + T2	28	14 (50.0)	14 (50.0)	1.000	0.537	0.537
T3 + T4	136	68 (50.0)	68 (50.0)			
Lymph node metastasis						
No	85	41 (48.2)	44 (51.8)	0.524	0.161	0.227
Yes	77	41 (53.4)	36 (46.6)			
Distant metastasis						
No	114	57 (50.0)	57 (50.0)	0.383	0.157	0.227
Yes	18	11 (61.1)	7 (38.9)			
Stage (UICC)						
I + II	83	40 (48.2)	43 (51.8)	0.417	0.071	0.227
III + IV	75	41 (54.7)	34 (45.3)			
Vascular invasion						
No	103	51 (49.5)	52 (50.5)	0.348	0.162	0.227
Yes	34	20 (58.8)	14 (41.2)			
Lymphatic invasion						
No	92	42 (45.7)	50 (54.3)	**0.027***	**0.010***	**0.070**
Yes	49	32 (65.3)	17 (34.7)			
Perineural invasion						
No	62	34 (54.8)	28 (45.2)	0.402	0.391	0.456
Yes	28	18 (64.3)	10 (35.7)			

Abbreviations: UICC, International Union Against Cancer; [a] Adjusted the effects of diagnosed age, gender, and location (colon and rectum); [b] Adjusted the effects of diagnosed age, gender, and location (colon and rectum), followed by Benjamini–Hochberg multiple correction; * A *p*-value of <0.05 was considered statistically significant.

Table S2. Over-representation analysis results of gene ontology (GO) biological process (BP) terms based on 18 NMF cluster-related genes.

GOBPID	*p* Value	Size	Term	Symbols
GO:0042391	1.33E-05	316	regulation of membrane potential	*CASQ2;DPP6;KCND2;RELN;SCN7A*
GO:0034765	1.95E-05	342	regulation of ion transmembrane transport	*CASQ2;DPP6;KCND2;RELN;SCN7A*

GO:0034762	2.43E-05	358	regulation of transmembrane transport	*CASQ2;DPP6;KCND2;RELN;SCN7A*
GO:0043269	0.000165	536	regulation of ion transport	*CASQ2;DPP6;KCND2;RELN;SCN7A*
GO:0001508	0.000198	112	action potential	*DPP6;KCND2;SCN7A*
GO:0035637	0.000209	114	multicellular organismal signaling	*CASQ2;DPP6;SCN7A*
GO:0019228	0.000434	30	neuronal action potential	*DPP6;SCN7A*
GO:0048265	0.000495	32	response to pain	*RELN;THBS4*
GO:0071804	0.000554	159	cellular potassium ion transport	*CASQ2;DPP6;KCND2*
GO:0071805	0.000554	159	potassium ion transmembrane transport	*CASQ2;DPP6;KCND2*
GO:0008038	0.000559	34	neuron recognition	*GAP43;NCAM2*
GO:0006813	0.001017	196	potassium ion transport	*CASQ2;DPP6;KCND2*
GO:1901379	0.001069	47	regulation of potassium ion transmembrane transport	*CASQ2;DPP6*
GO:0034220	0.0017	892	ion transmembrane transport	*CASQ2;DPP6;KCND2;RELN;SCN7A*
GO:0019226	0.001852	62	transmission of nerve impulse	*DPP6;SCN7A*
GO:0043266	0.001972	64	regulation of potassium ion transport	*CASQ2;DPP6*
GO:0015672	0.001982	540	monovalent inorganic cation transport	*CASQ2;DPP6;KCND2;SCN7A*
GO:0098662	0.002037	544	inorganic cation transmembrane transport	*CASQ2;DPP6;KCND2;SCN7A*
GO:1900273	0.002077	2	positive regulation of long-term synaptic potentiation	*RELN*
GO:0033555	0.002288	69	multicellular organismal response to stress	*RELN;THBS4*
GO:0098655	0.002875	598	cation transmembrane transport	*CASQ2;DPP6;KCND2;SCN7A*
GO:0034767	0.003061	80	positive regulation of ion transmembrane transport	*DPP6;RELN*
GO:0071603	0.003114	3	endothelial cell-cell adhesion	*THBS4*
GO:0097477	0.003114	3	lateral motor column neuron migration	*RELN*
GO:1900451	0.003114	3	positive regulation of glutamate receptor signaling pathway	*RELN*
GO:2000969	0.003114	3	positive regulation of alpha-amino-3-hydroxy-5-methyl-4-isoxazole propionate selective glutamate receptor activity	*RELN*
GO:0022607	0.003176	2074	cellular component assembly	*CASQ2;GAP43;KCND2;RELN;PDSS1;HMP19;RIC3*
GO:0098660	0.003471	630	inorganic ion transmembrane transport	*CASQ2;DPP6;KCND2;SCN7A*
GO:0034764	0.003528	86	positive regulation of transmembrane transport	*DPP6;RELN*
GO:0038026	0.00415	4	reelin-mediated signaling pathway	*RELN*
GO:0097475	0.00415	4	motor neuron migration	*RELN*
GO:0097476	0.00415	4	spinal cord motor neuron migration	*RELN*
GO:0044085	0.004977	2243	cellular component biogenesis	*CASQ2;GAP43;KCND2;RELN;PDSS1;HMP19;RIC3*
GO:0086029	0.005185	5	Purkinje myocyte to ventricular cardiac muscle cell signaling	*CASQ2*
GO:0086068	0.005185	5	Purkinje myocyte to ventricular cardiac muscle cell communication	*CASQ2*
GO:0097114	0.005185	5	N-methyl-D-aspartate receptor clustering	*RELN*
GO:1900271	0.005185	5	regulation of long-term synaptic potentiation	*RELN*
GO:0006461	0.00557	1171	protein complex assembly	*CASQ2;KCND2;PDSS1;HMP19;RIC3*
GO:0070271	0.005611	1173	protein complex biogenesis	*CASQ2;KCND2;PDSS1;HMP19;RIC3*
GO:0016198	0.006219	6	axon choice point recognition	*GAP43*

GO:0008037	0.006322	116	cell recognition	*GAP43;NCAM2*
GO:0030001	0.006864	763	metal ion transport	*CASQ2;DPP6;KCND2;SCN7A*
GO:0019800	0.007252	7	peptide cross-linking via chondroitin 4-sulfate glycosaminoglycan	*MAMDC2*
GO:0071313	0.007252	7	cellular response to caffeine	*CASQ2*
GO:0071415	0.007252	7	cellular response to purine-containing compound	*CASQ2*
GO:0097119	0.007252	7	postsynaptic density protein 95 clustering	*RELN*
GO:2001224	0.007252	7	positive regulation of neuron migration	*RELN*
GO:0055085	0.008142	1281	transmembrane transport	*CASQ2;DPP6;KCND2;RELN;SCN7A*
GO:0002097	0.008284	8	tRNA wobble base modification	*ADAT2*
GO:0021800	0.008284	8	cerebral cortex tangential migration	*RELN*
GO:0051451	0.008284	8	myoblast migration	*THBS4*
GO:0072578	0.008284	8	neurotransmitter-gated ion channel clustering	*RELN*
GO:0051899	0.008354	134	membrane depolarization	*RELN;SCN7A*
GO:0061003	0.009314	9	positive regulation of dendritic spine morphogenesis	*RELN*
GO:0097120	0.009314	9	receptor localization to synapse	*RELN*
GO:0043623	0.009317	430	cellular protein complex assembly	*CASQ2;HMP19;RIC3*
GO:0050731	0.009339	142	positive regulation of peptidyl-tyrosine phosphorylation	*RELN;THBS4*
GO:0090129	0.010344	10	positive regulation of synapse maturation	*RELN*
GO:1901017	0.010344	10	negative regulation of potassium ion transmembrane transporter activity	*CASQ2*
GO:0065003	0.010703	1368	macromolecular complex assembly	*CASQ2;KCND2;PDSS1;HMP19;RIC3*
GO:0032412	0.01132	157	regulation of ion transmembrane transporter activity	*CASQ2;RELN*
GO:0060315	0.011373	11	negative regulation of ryanodine-sensitive calcium-release channel activity	*CASQ2*
GO:0022898	0.011877	161	regulation of transmembrane transporter activity	*CASQ2;RELN*
GO:0010001	0.012161	163	glial cell differentiation	*GAP43;RELN*
GO:0006744	0.012401	12	ubiquinone biosynthetic process	*PDSS1*
GO:0021819	0.012401	12	layer formation in cerebral cortex	*RELN*
GO:0090128	0.012401	12	regulation of synapse maturation	*RELN*
GO:0006743	0.013428	13	ubiquinone metabolic process	*PDSS1*
GO:0048268	0.013428	13	clathrin coat assembly	*HMP19*
GO:0006811	0.01344	1446	ion transport	*CASQ2;DPP6;KCND2;RELN;SCN7A*
GO:0032409	0.01362	173	regulation of transporter activity	*CASQ2;RELN*
GO:0055065	0.014244	503	metal ion homeostasis	*CASQ2;SCN7A;RIC3*
GO:0005513	0.014454	14	detection of calcium ion	*CASQ2*
GO:0010649	0.014454	14	regulation of cell communication by electrical coupling	*CASQ2*
GO:0032793	0.014454	14	positive regulation of CREB transcription factor activity	*RELN*
GO:0090136	0.014454	14	epithelial cell-cell adhesion	*THBS4*
GO:1901380	0.014454	14	negative regulation of potassium ion transmembrane transport	*CASQ2*
GO:1901663	0.014454	14	quinone biosynthetic process	*PDSS1*
GO:0007158	0.015479	15	neuron cell-cell adhesion	*NCAM2*

GO:0031000	0.015479	15	response to caffeine	*CASQ2*
GO:2000310	0.015479	15	regulation of N-methyl-D-aspartate selective glutamate receptor activity	*RELN*
GO:0032879	0.015658	2102	regulation of localization	*CASQ2;DPP6;KCND2;RELN;SCN7A;THBS4*
GO:0050795	0.016107	189	regulation of behavior	*RELN;THBS4*
GO:0006812	0.016259	980	cation transport	*CASQ2;DPP6;KCND2;SCN7A*
GO:0048266	0.016502	16	behavioral response to pain	*THBS4*
GO:0050730	0.016593	192	regulation of peptidyl-tyrosine phosphorylation	*RELN;THBS4*
GO:0021801	0.017525	17	cerebral cortex radial glia guided migration	*RELN*
GO:0022030	0.017525	17	telencephalon glial cell migration	*RELN*
GO:0035418	0.017525	17	protein localization to synapse	*RELN*
GO:0060074	0.017525	17	synapse maturation	*RELN*
GO:0042063	0.01809	201	gliogenesis	*GAP43;RELN*
GO:0055080	0.018251	552	cation homeostasis	*CASQ2;SCN7A;RIC3*
GO:0043270	0.018431	203	positive regulation of ion transport	*DPP6;RELN*
GO:0051968	0.018547	18	positive regulation of synaptic transmission, glutamatergic	*RELN*
GO:0098771	0.019411	565	inorganic ion homeostasis	*CASQ2;SCN7A;RIC3*
GO:0051049	0.019414	1584	regulation of transport	*CASQ2;DPP6;KCND2;RELN;SCN7A*
GO:0010881	0.019568	19	regulation of cardiac muscle contraction by regulation of the release of sequestered calcium ion	*CASQ2*
GO:0086019	0.019568	19	cell-cell signaling involved in cardiac conduction	*CASQ2*
GO:0090023	0.019568	19	positive regulation of neutrophil chemotaxis	*THBS4*
GO:2000311	0.019568	19	regulation of alpha-amino-3-hydroxy-5-methyl-4-isoxazole propionate selective glutamate receptor activity	*RELN*
GO:2000463	0.019568	19	positive regulation of excitatory postsynaptic membrane potential	*RELN*
GO:0007413	0.020588	20	axonal fasciculation	*NCAM2*
GO:0060999	0.020588	20	positive regulation of dendritic spine development	*RELN*
GO:1901381	0.020588	20	positive regulation of potassium ion transmembrane transport	*DPP6*
GO:1902624	0.020588	20	positive regulation of neutrophil migration	*THBS4*
GO:0007204	0.021251	219	positive regulation of cytosolic calcium ion concentration	*CASQ2;RIC3*
GO:0032008	0.021607	21	positive regulation of TOR signaling	*RELN*
GO:1901020	0.021607	21	negative regulation of calcium ion transmembrane transporter activity	*CASQ2*
GO:1903170	0.021607	21	negative regulation of calcium ion transmembrane transport	*CASQ2*
GO:0061564	0.022047	593	axon development	*GAP43;NCAM2;RELN*
GO:0071822	0.022445	1643	protein complex subunit organization	*CASQ2;KCND2;PDSS1;HMP19;RIC3*
GO:0001941	0.022625	22	postsynaptic membrane organization	*RELN*
GO:0071624	0.022625	22	positive regulation of granulocyte chemotaxis	*THBS4*
GO:2001258	0.022625	22	negative regulation of cation channel activity	*CASQ2*
GO:0010882	0.023641	23	regulation of cardiac muscle contraction by calcium ion signaling	*CASQ2*
GO:0043267	0.023641	23	negative regulation of potassium ion transport	*CASQ2*

GO:0061001	0.023641	23	regulation of dendritic spine morphogenesis	*RELN*
GO:0008299	0.024657	24	isoprenoid biosynthetic process	*PDSS1*
GO:0050775	0.024657	24	positive regulation of dendrite morphogenesis	*RELN*
GO:0090022	0.024657	24	regulation of neutrophil chemotaxis	*THBS4*
GO:0051480	0.025013	239	cytosolic calcium ion homeostasis	*CASQ2;RIC3*
GO:0050801	0.025083	623	ion homeostasis	*CASQ2;SCN7A;RIC3*
GO:0010880	0.025672	25	regulation of release of sequestered calcium ion into cytosol by sarcoplasmic reticulum	*CASQ2*
GO:1902622	0.025672	25	regulation of neutrophil migration	*THBS4*
GO:0045838	0.026686	26	positive regulation of membrane potential	*RELN*
GO:2001222	0.026686	26	regulation of neuron migration	*RELN*
GO:0021511	0.027699	27	spinal cord patterning	*RELN*
GO:0060306	0.027699	27	regulation of membrane repolarization	*CASQ2*
GO:0060314	0.027699	27	regulation of ryanodine-sensitive calcium-release channel activity	*CASQ2*
GO:0007616	0.028711	28	long-term memory	*RELN*
GO:0010644	0.028711	28	cell communication by electrical coupling	*CASQ2*
GO:0021799	0.028711	28	cerebral cortex radially oriented cell migration	*RELN*
GO:0043268	0.028711	28	positive regulation of potassium ion transport	*DPP6*
GO:0060997	0.028711	28	dendritic spine morphogenesis	*RELN*
GO:0007271	0.029722	29	synaptic transmission, cholinergic	*RIC3*
GO:0014808	0.029722	29	release of sequestered calcium ion into cytosol by sarcoplasmic reticulum	*CASQ2*
GO:1900449	0.029722	29	regulation of glutamate receptor signaling pathway	*RELN*
GO:1903513	0.029722	29	endoplasmic reticulum to cytosol transport	*CASQ2*
GO:1903514	0.029722	29	calcium ion transport from endoplasmic reticulum to cytosol	*CASQ2*
GO:0007212	0.030732	30	dopamine receptor signaling pathway	*HMP19*
GO:0018149	0.030732	30	peptide cross-linking	*MAMDC2*
GO:1901661	0.030732	30	quinone metabolic process	*PDSS1*
GO:0008347	0.03174	31	glial cell migration	*RELN*
GO:1901016	0.03174	31	regulation of potassium ion transmembrane transporter activity	*CASQ2*
GO:2000273	0.03174	31	positive regulation of receptor activity	*RELN*
GO:0006935	0.032414	688	chemotaxis	*GAP43;RELN;THBS4*
GO:0042330	0.032414	688	taxis	*GAP43;RELN;THBS4*
GO:0007267	0.032487	1209	cell-cell signaling	*CASQ2;KCND2;RELN;RIC3*
GO:0070296	0.032748	32	sarcoplasmic reticulum calcium ion transport	*CASQ2*
GO:0071312	0.032748	32	cellular response to alkaloid	*CASQ2*
GO:0045214	0.033755	33	sarcomere organization	*CASQ2*
GO:0071622	0.033755	33	regulation of granulocyte chemotaxis	*THBS4*
GO:0086009	0.033755	33	membrane repolarization	*CASQ2*
GO:0010959	0.03395	282	regulation of metal ion transport	*CASQ2;DPP6*
GO:0042181	0.034761	34	ketone biosynthetic process	*PDSS1*

GO:0006936	0.03506	287	muscle contraction	*CASQ2;SCN7A*
GO:0007268	0.035507	713	synaptic transmission	*KCND2;RELN;RIC3*
GO:0061098	0.035766	35	positive regulation of protein tyrosine kinase activity	*RELN*
GO:0003008	0.035854	1854	system process	*CASQ2;DPP6;NCAM2;RELN;SCN7A*
GO:0050877	0.035874	1247	neurological system process	*DPP6;NCAM2;RELN;SCN7A*
GO:0051057	0.03677	36	positive regulation of small GTPase mediated signal transduction	*RELN*
GO:0051290	0.03677	36	protein heterotetramerization	*PDSS1*
GO:0051489	0.03677	36	regulation of filopodium assembly	*GAP43*
GO:0097061	0.03677	36	dendritic spine organization	*RELN*
GO:0007205	0.037773	37	protein kinase C-activating G-protein coupled receptor signaling pathway	*GAP43*
GO:0060291	0.037773	37	long-term synaptic potentiation	*RELN*
GO:0060998	0.037773	37	regulation of dendritic spine development	*RELN*
GO:0034622	0.039689	745	cellular macromolecular complex assembly	*CASQ2;HMP19;RIC3*
GO:0006901	0.039776	39	vesicle coating	*HMP19*
GO:0051208	0.039776	39	sequestering of calcium ion	*CASQ2*
GO:0018108	0.040586	311	peptidyl-tyrosine phosphorylation	*RELN;THBS4*
GO:0006400	0.040776	40	tRNA modification	*ADAT2*
GO:0032413	0.040776	40	negative regulation of ion transmembrane transporter activity	*CASQ2*
GO:0018212	0.041061	313	peptidyl-tyrosine modification	*RELN;THBS4*
GO:0030335	0.041538	315	positive regulation of cell migration	*RELN;THBS4*
GO:0043113	0.041775	41	receptor clustering	*RELN*
GO:0051926	0.041775	41	negative regulation of calcium ion transport	*CASQ2*
GO:0021795	0.042773	42	cerebral cortex cell migration	*RELN*
GO:2000147	0.043224	322	positive regulation of cell motility	*RELN;THBS4*
GO:0086010	0.043771	43	membrane depolarization during action potential	*SCN7A*
GO:1900006	0.043771	43	positive regulation of dendrite development	*RELN*
GO:0006874	0.043955	325	cellular calcium ion homeostasis	*CASQ2;RIC3*
GO:0055078	0.044767	44	sodium ion homeostasis	*SCN7A*
GO:0060996	0.044767	44	dendritic spine development	*RELN*
GO:0086065	0.044767	44	cell communication involved in cardiac conduction	*CASQ2*
GO:0051272	0.04543	331	positive regulation of cellular component movement	*RELN;THBS4*
GO:0042246	0.045762	45	tissue regeneration	*GAP43*
GO:0003012	0.046674	336	muscle system process	*CASQ2;SCN7A*
GO:0051966	0.046756	46	regulation of synaptic transmission, glutamatergic	*RELN*
GO:0055074	0.046924	337	calcium ion homeostasis	*CASQ2;RIC3*
GO:0040017	0.047426	339	positive regulation of locomotion	*RELN;THBS4*
GO:0021517	0.047749	47	ventral spinal cord development	*RELN*
GO:0072503	0.048183	342	cellular divalent inorganic cation homeostasis	*CASQ2;RIC3*
GO:0032410	0.048741	48	negative regulation of transporter activity	*CASQ2*

Table S3. Signaling pathway impact analysis (SPIA) results in CRC patients.

Name	KEGG ID	Set size [a]	$PERT_{Total}$ [b]	P_{PERT}	Status [c]
Staphylococcus aureus infection	hsa5150	49	−22.98251156	2×10^{-6}	Inhibited
Extracellular matrix-receptor interaction	hsa4512	81	−33.197358	2×10^{-6}	Inhibited
Focal adhesion	hsa4510	192	−97.61084388	2×10^{-6}	Inhibited
Basal cell carcinoma	hsa5217	50	−26.2231231	2×10^{-6}	Inhibited
Regulation of actin cytoskeleton	hsa4810	187	−45.08424287	2×10^{-6}	Inhibited
Cytokine-cytokine receptor interaction	hsa4060	212	−21.18470548	2×10^{-6}	Inhibited
Cholinergic synapse	hsa4725	96	−23.06809223	2×10^{-6}	Inhibited
Chemokine signaling pathway	hsa4062	173	−46.98870421	2×10^{-6}	Inhibited
Calcium signaling pathway	hsa4020	151	−34.10535107	2×10^{-6}	Inhibited
Pathways in cancer	hsa5200	304	−66.39495865	2×10^{-6}	Inhibited

[a] Number of genes on the corresponding pathway. [b] The observed total perturbation accumulation in the pathway.
[c] Direction in which the pathway is perturbed. Significant KEGG pathways are shown (*p*-value: $< 2 \times 10^{-6}$).

Table S4. Correlations between clinic pathological features and *let-7g* expression ratios for 20 postoperative Taiwanese colorectal cancer patients.

Characteristics	Total Cases	*let-7g* Expression Ratio		Logistic *p*-Value [a]	Logistic *p*-Value Adjusted by Covariates [b]	Fisher's Exact *p*-Value [a]
	N	Low N (%)	High N (%)			
Gender						
Female	14	9 (64.3)	5 (35.7)	0.374		0.141
Male	6	1 (16.7)	5 (83.3)			
Age (years)						
<median	10	4 (40.0)	6 (60.0)	0.410		0.656
≥median	10	6 (60.0)	4 (40.0)			
Tumor size						
<5 cm	13	8 (61.5)	5 (38.5)	0.210	0.337	0.350
≥5 cm	7	2 (28.6)	5 (71.4)			
Depth of tumor invasion						
T1 + T2	5	3 (60.0)	2 (40.0)	0.186	0.870	1.000
T3 + T4	15	7 (46.7)	8 (53.3)			
Lymph node metastasis						
No	10	6 (60.0)	4 (40.0)	0.117	0.490	0.656
Yes	10	4 (40.0)	6 (60.0)			
Stage (UICC)						
I + II	10	6 (60.0)	4 (40.0)	0.117	0.490	0.656
III + IV	10	4 (40.0)	6 (60.0)			
Histology						
MD	18	10 (55.6)	8 (44.4)	0.995	0.995	0.474
PD	2	0 (0.0)	2 (100.0)			
Vascular invasion						
No	14	7 (50.0)	7 (50.0)	0.374	0.573	1.000
Yes	6	3 (50.0)	3 (50.0)			
Perineural invasion						
No	17	10 (58.8)	7 (41.2)	0.996	0.996	0.211
Yes	3	0 (0.0)	3 (100.0)			

Abbreviations: MD, moderately well differentiated; PD, poorly differentiated; UICC, International Union Against Cancer; [a] A *p*-value of <0.05 was considered statistically significant. [b] Adjusted for the effects of age and sex.

© 2019 by the authors. Licensee MDPI, Basel, Switzerland. This article is an open access article distributed under the terms and conditions of the Creative Commons Attribution (CC BY) license (http://creativecommons.org/licenses/by/4.0/).
